# Supplementary material for: Density functionals with asymptotic-potential corrections are required for the simulation of spectroscopic properties of materials
Source: Chem Sci. 2021 Dec 31;13(5):1492–503. doi: 10.1039/d1sc03738b (PMC8809424; doi:10.1039/d1sc03738b)
Supplement: SC-013-D1SC03738B-s001 [file SC-013-D1SC03738B-s001.pdf]

Electronic Supplementary Material (ESI) for Chemical Science.
This journal is © The Royal Society of Chemistry 2021

Density functionals with asymptotic-potential corrections are required for the simulation of spectroscopic properties of materials.

Musen Li,[a] Rika Kobayashi,[b] Roger D. Amos,[b] Michael J. Ford[c] and Jeffrey R. Reimers[a,c]
a International Centre for Quantum and Molecular Structures and Department of Physics, Shanghai University, Shanghai 200444, China.
b: ANU Supercomputer Facility, Leonard Huxley Bldg. 56, Mills Rd, Canberra, ACT, 2601, Australia.
c: University of Technology Sydney, School of Mathematical and Physical Sciences, Ultimo, New South Wales 2007, Australia.
Email: Jeffrey.Reimers@uts.edu.au, Mike.Ford@uts.edu.au

Electronic Supporting Information

Table S1. Comparison of observed and calculated lattice parameters (in Å).

Material	obs.	CAM-B3LYP[1]	B3LYP[b]	HSE06[1-3]	DD-RSC-CAM[2]	PBE[2, 3]	SCAN[4]
Al_2O_3	4.7589 [5]	4.762[a]					
AlAs	5.6614 [6]	5.664[a]					
AlN-wz	3.1111 [6]	3.099[a]					3.090
AlP-zb	5.451 [3]	5.458[a]		2.543			5.482
BN	2.5044 [7]	2.506	2.493	2.491	2.534	2.543	2.551
BP	4.5383 [6]	4.538[a]		4.421	4.527	4.521	
C	3.5669 [8]	3.5671	3.559	3.548	3.552	3.548	3.556
CaO	4.811 [6]	4.813[a]		4.796		4.828	
GaAs	5.6536 [6]	5.658[a]		5.684		5.747	
GaN-zb	4.509 [3]	4.506[a]		4.457			4.499
GaP-zb	5.439 [3]	5.447[a]		5.449			5.446
Ge	5.6579 [6]	5.660[a]			5.719		
InAs	6.0583 [6]	6.063[a]					
InP	5.8687 [6]	5.875[a]					
InSb	6.4794 [6]	6.478[a]					
LiCl	5.070 [3]	5.067[a]		5.147	5.092		5.096
LiF	3.972 [3]	3.968[a]		4.018	3.927	4.060	3.986
MgO	4.217 [9]	4.2155	4.207	4.210	4.158	4.254	4.204
NaCl	5.6402 [10, 11]	5.6472	5.670	5.662	5.594	5.698	5.593
SiC-3c	4.346 [3]	4.347[a]		4.351	4.349	4.380	4.360
Si	5.431 [12]	5.4360	5.472	5.442	5.447	5.470	5.434
SiO_2	4.9124 [13]	4.909[a]					
MAD		0.004	0.020	0.033	0.031	0.044	0.021

a: this work.
b: see also [14]

Table S2. Calculated and observed orbital bandgap, in eV.

Material	Obs.	PBE [2,3]	SCAN [4,15,16]	mBJLDA [4]	PBE0 [16,17]	HSE06 [1-3]	B3LYP [a]	CAM-B3LYP	WOT-SRSH [18]	PBE0(α) [19]	sc-hybrid [17]	RSH m_{WS} [17]	RSH m_{TF} [17]	RSH $m_{erfc\text{-}fit}$ [17]	DD-PBEH [2]	RS-DDH [2]	DD0-RHS-CAM [2]	DD-RSH-CAM [2]
Al_2O_3-α	9.57 [20,21]	6.27		8.34	8.80	8.07		9.42			9.71	9.63	9.61		9.40	9.61	9.60	9.51
AlAs	2.23 [6]	1.32	1.74	2.13		1.93		2.69							1.91	1.90	2.05	2.11
AlN-wz	6.23 [22]	4.18	3.97	5.51	6.31	4.55		6.41	6.60	7.30	6.23	6.22	6.23		6.10	6.11	6.25	6.26
AlP-zb	2.45 [22]	1.56	1.92	2.31	2.98	2.33		2.99	2.60		2.37	2.43	2.42	2.40	2.30	2.33	2.41	2.52
h-BN	6.42 [23]	4.29	4.78		6.30	5.58		6.62 [1]										
BN	6.36 [24]	4.53	4.98	5.8	6.51	5.79	5.93				6.33	6.33	6.34	6.33	6.37	6.39	6.55	
BP	2.10 [25 b]	1.25	1.54	1.85		1.99		2.58							1.92	1.93	2.02	2.02
C	5.50 [32]	4.21	4.54	4.92	5.95	5.30 [1]	5.99	5.66 [1]	5.7	5.7	5.42	5.44	5.45	5.43	5.56	5.57	5.63	5.61
CaO	7.09 [26]	3.66		5.35		5.30		7.48		6.29					6.42	6.39	6.99	7.17
GaAs	1.52 [22]	0.42	0.77	1.64		1.26		1.72							0.79	0.90	1.38	1.45
GaN-zb	3.51 [22]	1.94	1.96	2.85	3.68	2.92		3.50	3.80		3.26	3.30	3.30		3.32	3.34	3.43	3.50
GaP-zb	2.35 [22]	1.65	1.83	2.25	2.93	2.30		3.09	2.40						2.15	2.19	2.36	2.42
Ge	0.74 [22]	metal	0.14	0.83		0.61		1.05							0.18	0.22	0.34	0.50
InAs	0.42 [6]		metal	0.67				0.65										
InP	1.42 [6 b]	0.69	1.02	2.31		1.47		1.58							1.21	1.25	1.49	1.55
InSb	0.24 [6 b]		metal	0.37				0.65										
LiCl	9.40 [27]	6.41	7.33	8.64	8.66	7.78		9.65			9.62	9.52	9.54		9.49	9.41	9.87	9.89
LiF	14.2 [28]	9.27	10.10	12.89	12.18	11.47		13.96	15.40		15.69	15.24	15.18	15.42	14.90	14.59	15.97	15.56
MgO	7.67 [29,30]	4.8	5.79	7.13	7.25	6.46 [1]	6.97	7.54 [1]	8.2	7.71	8.33	8.23	8.22	8.27	7.93	7.88	8.28	8.19
NaCl	8.6 [31]	5.2	5.99	8.45	7.26	6.43 [1]	6.78	8.83 [1]		8.71	8.84	8.6	8.66	8.71	8.75	8.64	9.1	9.1
Si	1.17 [32]	0.7	0.82	1.15	1.75	1.16 [1]	1.78	2.26 [1]	1.1	1.56	0.99	1.03	1.02	1.01	0.96	0.99	1.1	1.14
SiC-3c	2.42 [33]	1.36	1.72	2.25	2.91	2.26		3.18		2.40	2.29	2.32	2.32	2.31	2.33	2.35	2.41	2.47
SiO_2	9.65 [34]	5.95		8.70		7.69		9.77		10.20					9.99	9.88	10.58	10.40
MAD		2.01	1.30	0.52	0.60	0.86	0.81	0.33	0.35	0.40	0.30	0.20	0.20	0.30	0.28	0.23	0.30	0.25
MAX		4.93	4.10	1.75	2.02	2.73	1.82	1.09	1.20	1.07	1.49	1.04	0.98	1.22	0.73	0.71	1.77	1.36

a: this work, see also.[35] b: uncorrected optical bandgap.

Table S1 and S2 References

1 M. Li, J. R. Reimers, M. J. Ford, R. Kobayashi and R. D. Amos, *J. Comput. Chem.*, 2021, **42**, 1486.

2 W. Chen, G. Miceli, G.-M. Rignanese and A. Pasquarello, *Phys. Rev. Mater.*, 2018, **2**, 073803.

3 L. Schimka, J. Harl and G. Kresse, *J. Chem. Phys.*, 2011, **134**, 024116.

4 F. Tran, P. Kovács, L. Kalantari, G. K. H. Madsen and P. Blaha, *J. Chem. Phys.*, 2018, **149**, 144105.

5 R. E. Newnham and Y. M. de Haan, *Z. Krist.* , 1962, **117**, 235.

6 O. Madelung, *Semiconductors: Data Handbook*, Springer-Verlag, Berlin, 2004.

7 W. Paszkowicz, J. B. Pelka, M. Knapp, T. Szyszko and S. Podsiadlo, *Applied Physics A*, 2002, **75**, 431.

8 S. Shikata, T. Tanno, T. Teraji, H. Kanda, T. Yamada and J.-i. Kushibiki, *Japanese Journal of Applied Physics*, 2018, **57**, 111301.

9 B. Zaidi, S. Belghit, M. S. Ullah, B. Hadjoudja, A. Guerraoui, S. Gagui, N. Houaidji, B. Chouial and C. Shekhar, *Metallofiz. Noveishie Tekhnol.*, 2019, **41**, 1121—1126

10 W. T. Barrett and W. E. Wallace, *J. Am. Chem. Soc.*, 1954, **76**, 366.

11 V. S. Urusov and V. V. Blinov, *Bulletin of the Academy of Sciences of the USSR, Division of chemical science*, 1970, **19**, 231.

12 J. R. Rumble, T. J. Bruno, M. J. Doa and C. R. C. Press, *CRC handbook of chemistry and physics a ready-reference book of chemical and physical data*, CRC Press, Boco Raton, 2020.

13 R. Ratnawulan, A. Fauzi and A. E. S. Hayati, *IOP Conference Series: Materials Science and Engineering*, 2018, **335**, 012008.

14 J. Paier, M. Marsman and G. Kresse, *J. Chem. Phys.*, 2007, **127**, 024103.

15 D. Mejia-Rodriguez and S. B. Trickey, *Phys. Rev. B*, 2018, **98**, 115161.

16 P. Borlido, T. Aull, A. W. Huran, F. Tran, M. A. L. Marques and S. Botti, *J. Chem. Theory Comput.*, 2019, **15**, 5069.

17 J. H. Skone, M. Govoni and G. Galli, *Phys. Rev. B*, 2016, **93**, 235106.

18 D. Wing, G. Ohad, J. B. Haber, M. R. Filip, S. E. Gant, J. B. Neaton and L. Kronik, *Proceedings of the National Academy of Sciences*, 2021, **118**, e2104556118.

19 G. Miceli, W. Chen, I. Reshetnyak and A. Pasquarello, *Phys. Rev. B*, 2018, **97**, 121112.

20 R. H. French, D. J. Jones and S. Loughin, *J. Am. Ceram. Soc.*, 1994, **77**, 412.

21 F. G. Will, H. G. DeLorenzi and K. H. Janora, *J. Am. Ceram. Soc.*, 1992, **75**, 295.

22 I. Vurgaftman, J. R. Meyer and L. R. Ram-Mohan, *J. Appl. Phys.*, 2001, **89**, 5815.

23 T. C. Doan, J. Li, J. Y. Lin and H. X. Jiang, *Appl. Phys. Lett.*, 2016, **109**, 122101.

24 D. A. Evans, A. G. McGlynn, B. M. Towlson, M. Gunn, D. Jones, T. E. Jenkins, R. Winter and N. R. J. Poolton, *Journal of Physics Condensed Matter*, 2008, **20**.

25 A. Agui, S. Shin and Y. Kumashiro, *J. Phys. Soc. Jpn.*, 1999, **68**, 166.

26 R. C. Whited, C. J. Flaten and W. C. Walker, *Solid State Commun.*, 1973, **13**, 1903.

27 S.-i. Nakai and T. Sagawa, *J. Phys. Soc. Jpn.*, 1969, **26**, 1427.

28 M. Piacentini, D. W. Lynch and C. G. Olson, *Phys. Rev. B*, 1976, **13**, 5530.

29 L. X. Benedict, E. L. Shirley and R. B. Bohn, *Phys. Rev. Lett.*, 1998, **80**, 4514.

30 U. Schönberger and F. Aryasetiawan, *Phys. Rev. B*, 1995, **52**, 8788.

31 F. Bechstedt, K. Seino, P. H. Hahn and W. G. Schmidt, *Phys. Rev. B*, 2005, **72**, 245114.

32 M. S. Hybertsen and S. G. Louie, *Phys. Rev. B*, 1986, **34**, 5390.

33 R. G. Humphreys, D. Bimberg and W. J. Choyke, *Solid State Commun.*, 1981, **39**, 163.

34 H. R. Philipp, *Solid State Commun.*, 1966, **4**, 73.

35 J. Muscat, A. Wander and N. M. Harrison, *Chem. Phys. Lett.*, 2001, **342**, 397.

Table S3. Bandgaps for the new materials CAM-B3LYP calculations listed in Table S2 as a function of the sampling of the Brillion zone.

Material	*k*-points	number	bandgap / eV	Material	*k*-points	number	bandgap / eV
Al_2O_3	2x2x1	4	9.630	AlAs	2×2×2	8	2.923
	4x4x2	32	9.474		4×4×4	64	2.905
	8x8x4	256	9.422		6×6×6	216	2.878
	9x9x5	405	9.421		8×8×8	512	2.766
	12x12x5	720	9.416		10×10×10	1000	2.732
	12x12x6	864	9.416		12×12×12	1728	2.714
					15×15×15	3375	2.709
AlN-wz	2×2×1	4	6.570	AlP-zb	2×2×2	8	3.132
	4×4×2	32	6.465		4×4×4	64	3.096
	6×6×3	108	6.430		6×6×6	216	3.073
	8×8×4	256	6.417		8×8×8	512	3.066
	10×10×4	400	6.415		12×12×12	1728	3.065
	12×12×5	720	6.412				
	15×15×6	1350	6.410				
	16×16×6	1536	6.410				
BP	2×2×2	8	2.793	CaO	2×2×2	8	7.713
	4×4×4	64	2.775		4×4×4	64	7.572
	6×6×6	216	2.748		6×6×6	216	7.509
	8×8×8	512	2.636		8×8×8	512	7.493
	10×10×10	1000	2.597		10×10×10	1000	7.487
	12×12×12	1728	2.588		12×12×12	1728	7.484
	15×15×15	3375	2.586		15×15×15	3375	7.483
GaAs	2×2×2	8	2.331	GaN-zb	2×2×2	8	3.717
	4×4×4	64	1.996		4×4×4	64	3.603
	6×6×6	216	1.870		6×6×6	216	3.537
	8×8×8	512	1.799		8×8×8	512	3.512
	10×10×10	1000	1.791		10×10×10	1000	3.501
	12×12×12	1728	1.787		12x12x12	1728	3.498
	15×15×15	3375	1.786		15x15x15	3375	3.496
GaP-zb	2×2×2	8	3.287	Ge	2×2×2	8	1.477

	4×4×4	64	3.210		4×4×4	64	1.189
	6×6×6	216	3.252		6×6×6	216	1.007
	8×8×8	512	3.130		8×8×8	512	0.999
	10×10×10	1000	3.093		10×10×10	1000	1.503
	12×12×12	1728	3.090		12×12×12	1728	0.989
InAs	2×2×2	8	1.092	InP	2×2×2	8	1.745
	4×4×4	64	0.837		4×4×4	64	1.683
	6×6×6	216	0.690		6×6×6	216	1.618
	8×8×8	512	0.662		8×8×8	512	1.593
	10×10×10	1000	0.660		10×10×10	1000	1.586
	12×12×12	1728	0.659		12×12×12	1728	1.584
	15×15×15	3375	0.658		15×15×15	3375	1.579
InSb	2×2×2	8	1.042	LiCl	4×4×4	64	9.743
	4×4×4	64	0.814		5×5×5	125	9.703
	6×6×6	216	0.661		6×6×6	216	9.684
	8×8×8	512	0.580		7×7×7	343	9.674
	10×10×10	1000	0.559		8×8×8	512	9.669
	12×12×12	1728	0.553		9×9×9	729	9.665
	15×15×15	3375	0.551		10×10×10	1000	9.660
					11×11×11	1331	9.657
					12×12×12	1728	9.656
					13×13×13	2197	9.655
					14×14×14	2744	9.654
LiF	4×4×4	64	14.030	SiC	4×4×4	64	3.225
	5×5×5	125	13.995		5×5×5	125	3.692
	6×6×6	216	13.979		6×6×6	216	3.196
	7×7×7	343	13.971		7×7×7	343	3.437
	8×8×8	512	13.967		8×8×8	512	3.184
	9×9×9	729	13.964		9×9×9	729	3.332
	10×10×10	1000	13.962		10x10x10	1000	3.179
	11×11×11	1331	13.961		12x12x12	1728	3.177
	12×12×12	1728	13.960		14x14x14	2744	3.176
	13×13×13	2197	13.958		16x16x16	4096	3.175

	14×14×14	2744	13.958
	15×15×15	3375	13.957
SiO_2	2x2x1	4	9.903
	4x4x2	32	9.770
	8x8x4	256	9.744
	9x9x5	405	9.739
	12x12x5	720	9.738
	12x12x6	864	9.738

Table S4. Details of calculations of the CAM-B3LYP optical transition energies for Al_2O_3 and LiF.[a]

Material	*k*-points	ENCUT / eV	orbital bandgap / eV	exciton binding energy / eV	optical transition energy / eV
LiF	8×8×8	400	13.96	1.36	12.60
	8×8×8	850	13.97	1.30	12.67
Al_2O_3	2×2×1	400	9.42	0.34	9.08
	4×4×2	850	9.47	0.30	9.17
	9×9×5	850	9.42	0.25	9.17
	12×12×6	850	9.42	0.21	9.21

a: calculations in the table are for the lowest triplet state. The singlet-triplet difference is estimated as twice the energy difference between excited-state calculations with parallel spins (triplet) and with antiparallel spins (half singlet, half triplet). For Al_2O_3, the singlet-triplet difference is 0.011 eV using 4×4×2 *k*-points and 0.010 eV using 9×9×5. For LiF, the singlet-triplet difference is 0.008 eV at 8×8×8 *k*-points. Hence the best-estimate singlet excitation energies are 12.68 eV for LiF and 9.22 eV for Al_2O_3.

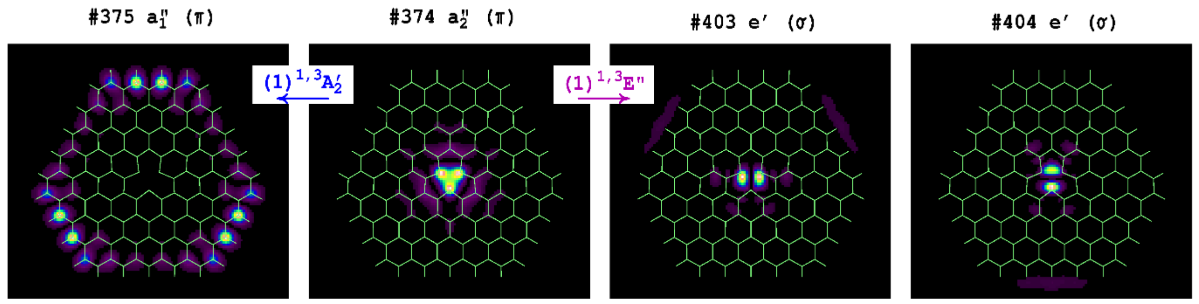

Fig. S1. Key HSE06 orbitals of **4**, analogous to the CAM-B3LYP orbitals shown in Fig. 2.

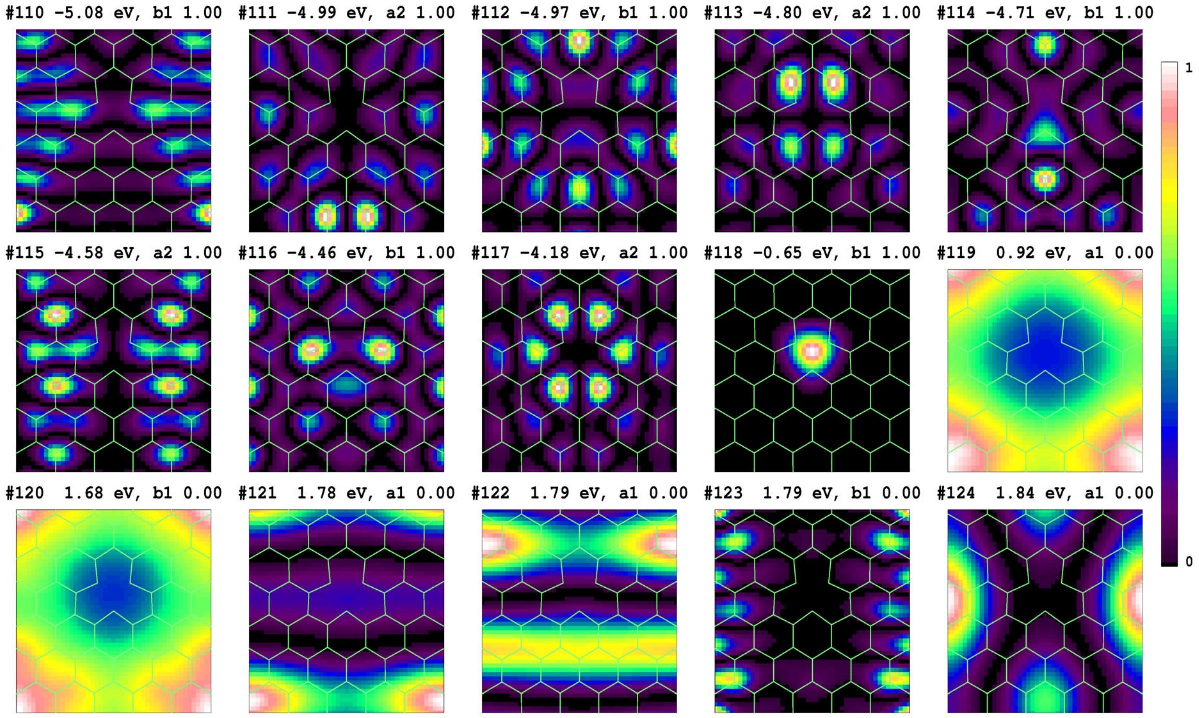

Fig. S2. Frontier orbitals from HSE06, $(1)^1A_1$ state, **P₅₃** 1×1×1 model, showing the relative electron density at a distance of 0.80 Å above the h-BN plane.

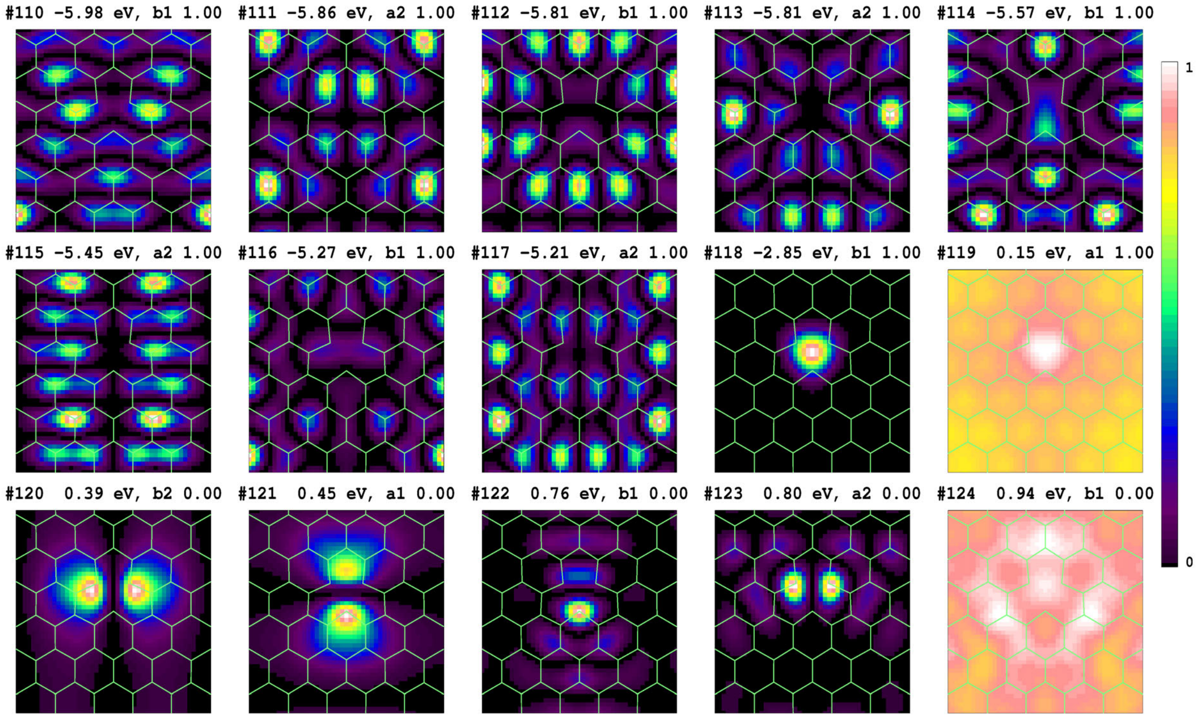

Fig. S3. Frontier orbitals from HSE06, $(1)^3B_1$ state, **P₅₃** 1×1×1 model, showing the relative electron density at a distance of 0.80 Å above the h-BN plane.

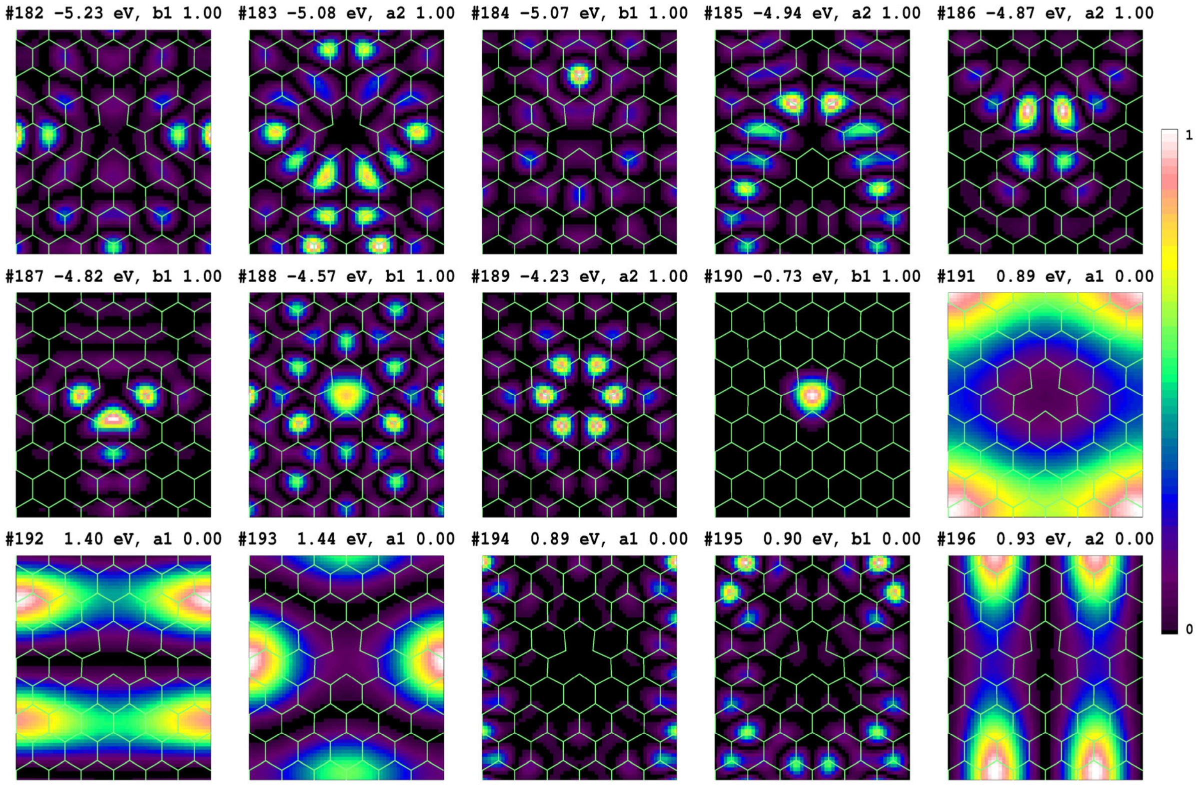

Fig. S4. Frontier orbitals from HSE06, $(1)^1A_1$ state, $\mathbf{P}_{64}$ 1×1×1 model, showing the relative electron density at a distance of 0.80 Å above the h-BN plane.

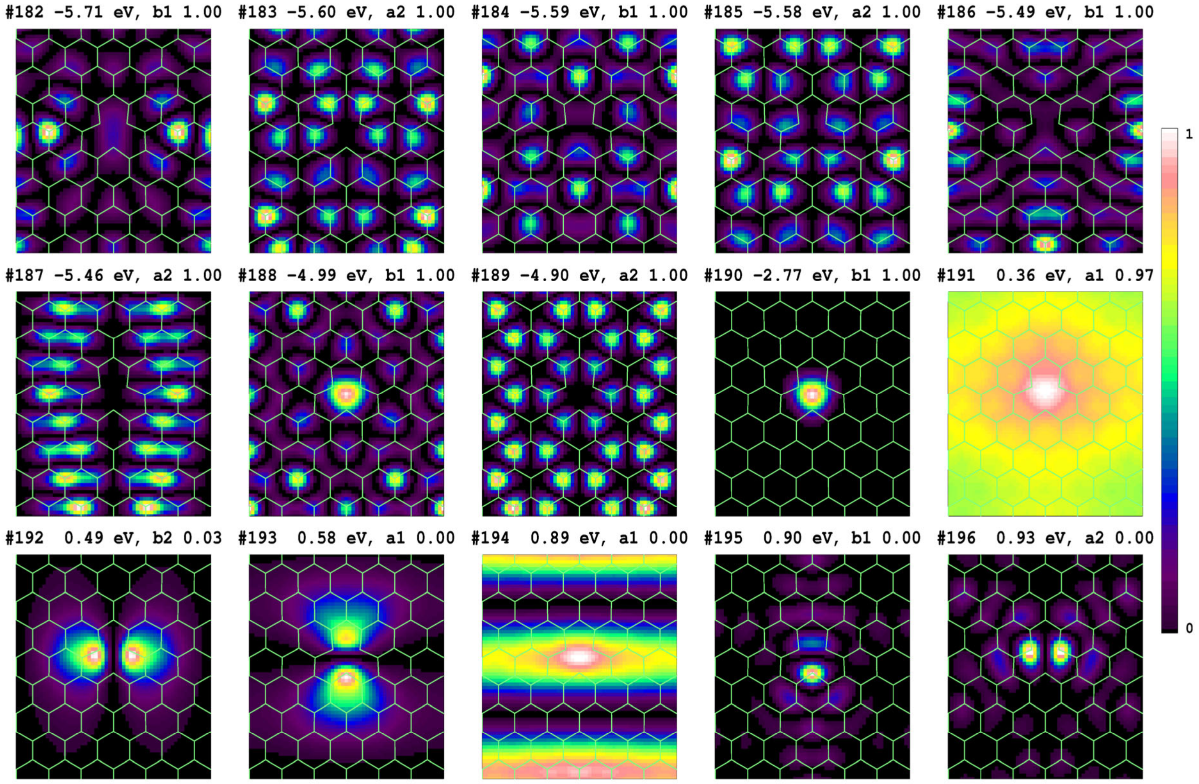

Fig. S5. Frontier orbitals from HSE06, $(1)^3B_1$ state, $\mathbf{P}_{64}$ 1×1×1 model, showing the relative electron density at a distance of 0.80 Å above the h-BN plane.

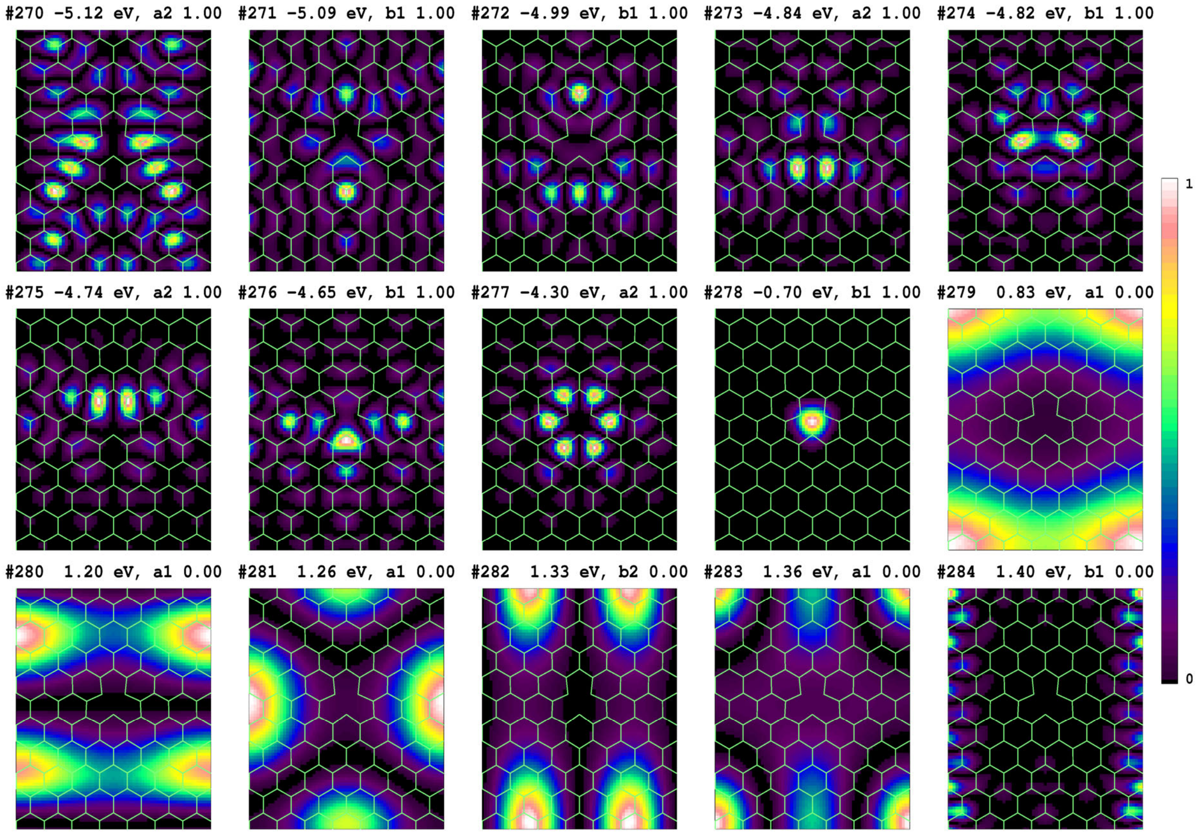

Fig. S6. Frontier orbitals from HSE06, $(1)^1A_1$ state, $\mathbf{P}_{75}$ 1×1×1 model, showing the relative electron density at a distance of 0.80 Å above the h-BN plane.

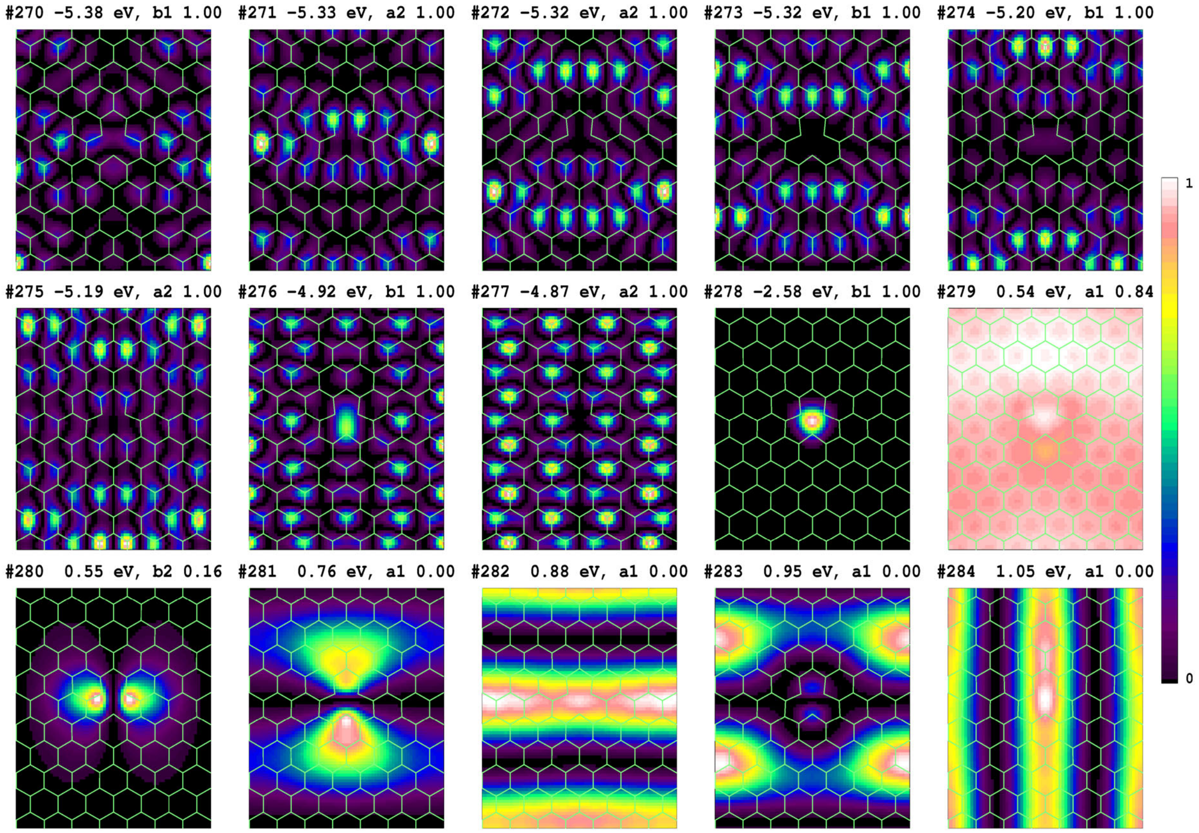

Fig. S7. Frontier orbitals from HSE06, $(1)^3B_1$ state, $\mathbf{P}_{75}$ 1×1×1 model, showing the relative electron density at a distance of 0.80 Å above the h-BN plane.

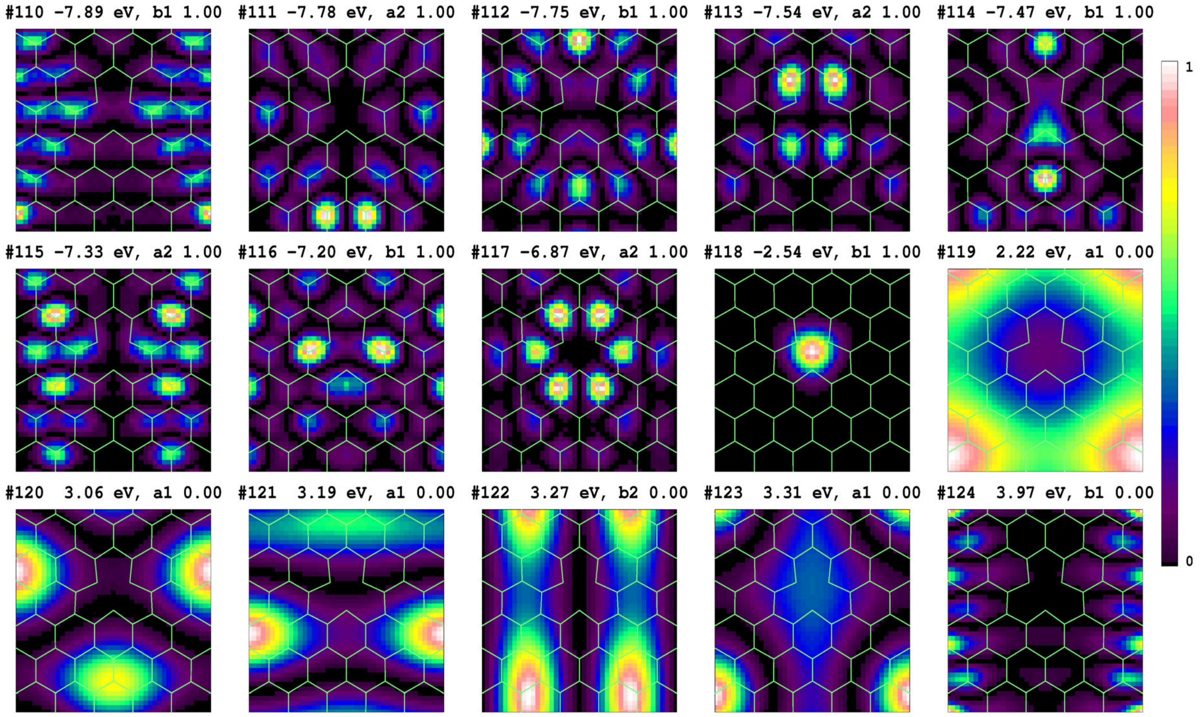

Fig. S8. Frontier orbitals from CAM-B3LYP, $(1)^1A_1$ state, $\mathbf{P_{53}}$ 1×1×1 model, showing the relative electron density at a distance of 0.80 Å above the h-BN plane.

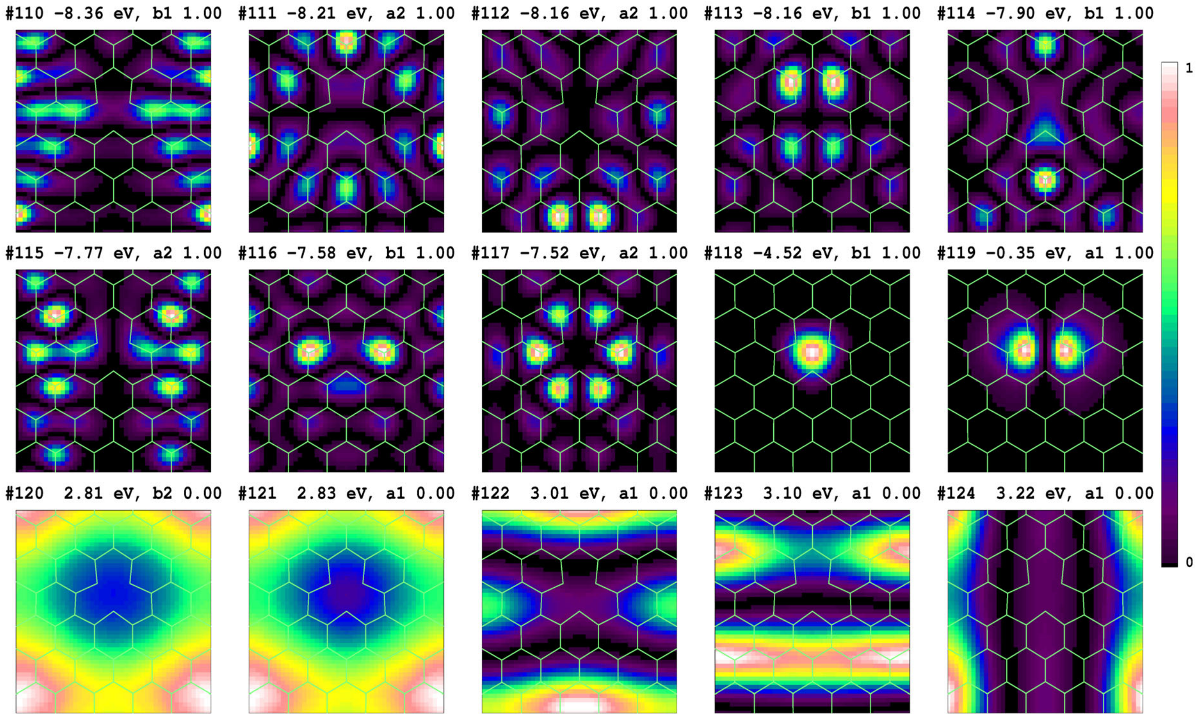

Fig. S9. Frontier orbitals from CAM-B3LYP, $(1)^3B_1$ state, $\mathbf{P_{53}}$ 1×1×1 model, showing the relative electron density at a distance of 0.80 Å above the h-BN plane.

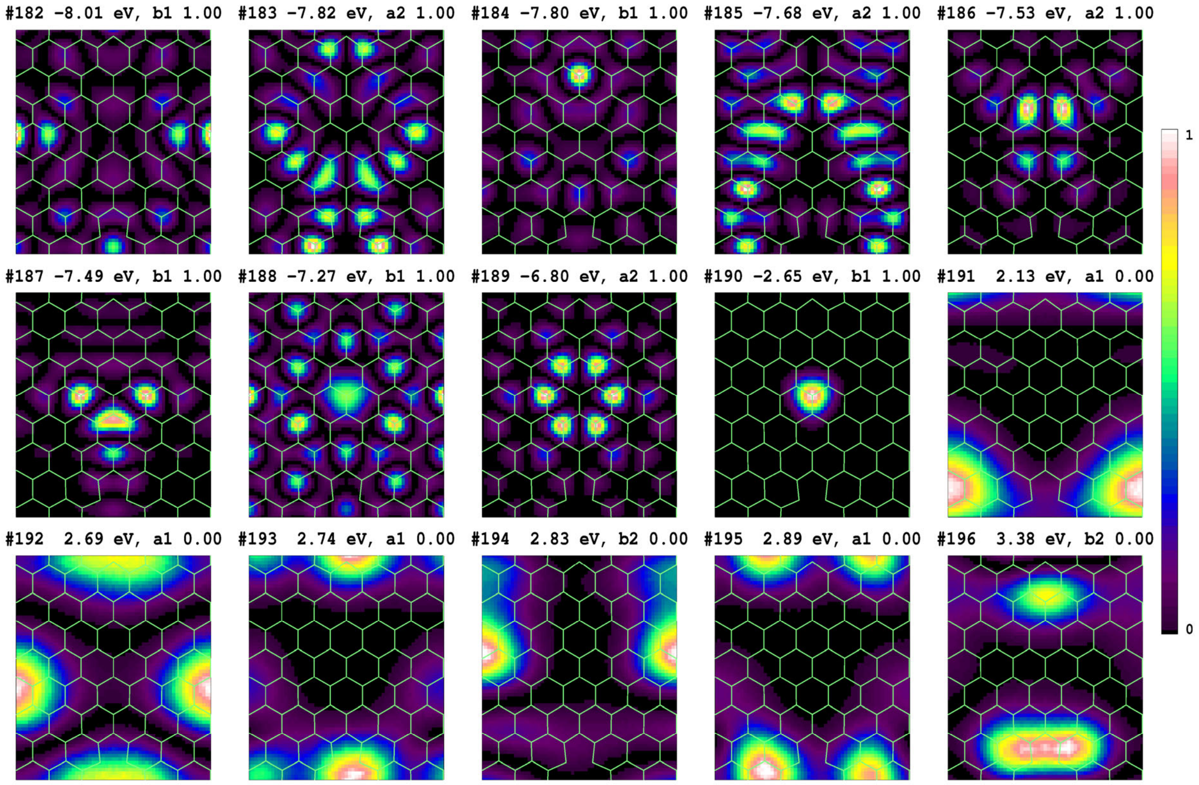

Fig. S10. Frontier orbitals from CAM-B3LYP, $(1)^1A_1$ state, **P$_{64}$** 1×1×1 model, showing the relative electron density at a distance of 0.80 Å above the h-BN plane.

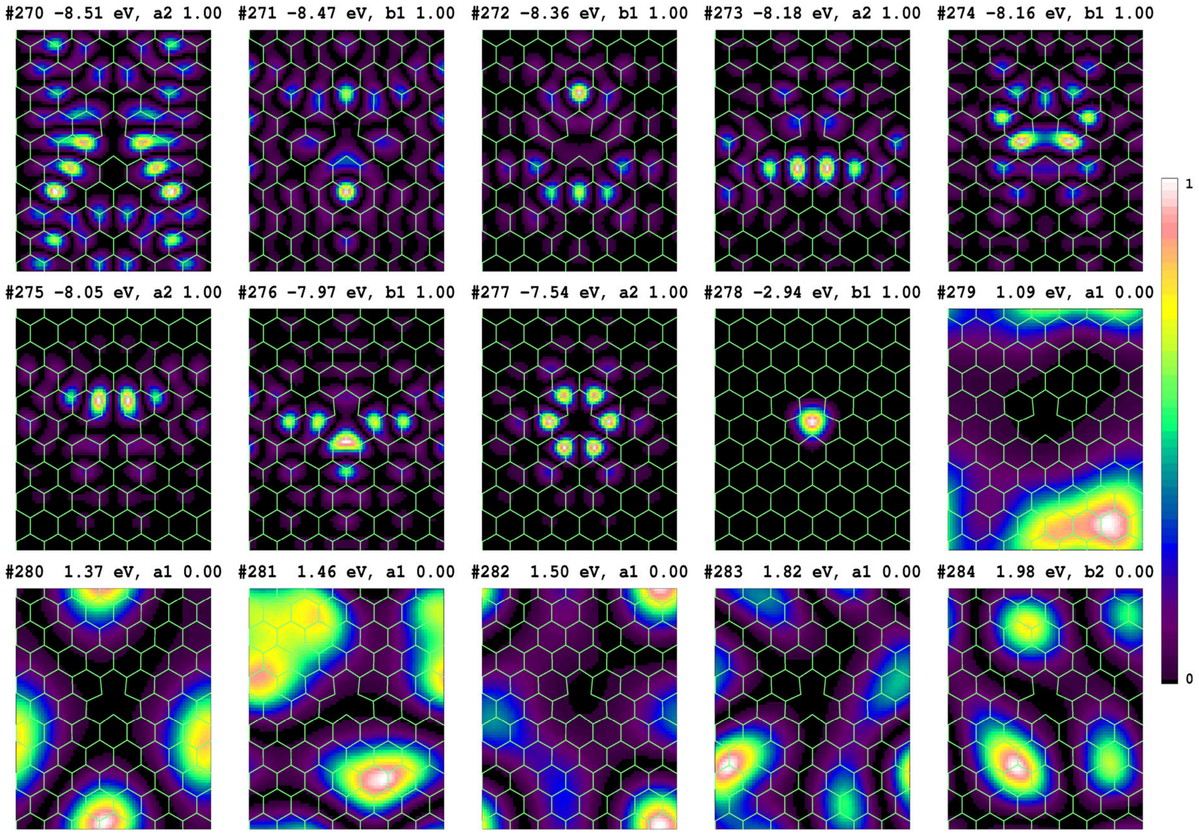

Fig. S11. Frontier orbitals from CAM-B3LYP, $(1)^1A_1$ state, **P**$_{75}$ 1×1×1 model, showing the relative electron density at a distance of 0.80 Å above the h-BN plane.

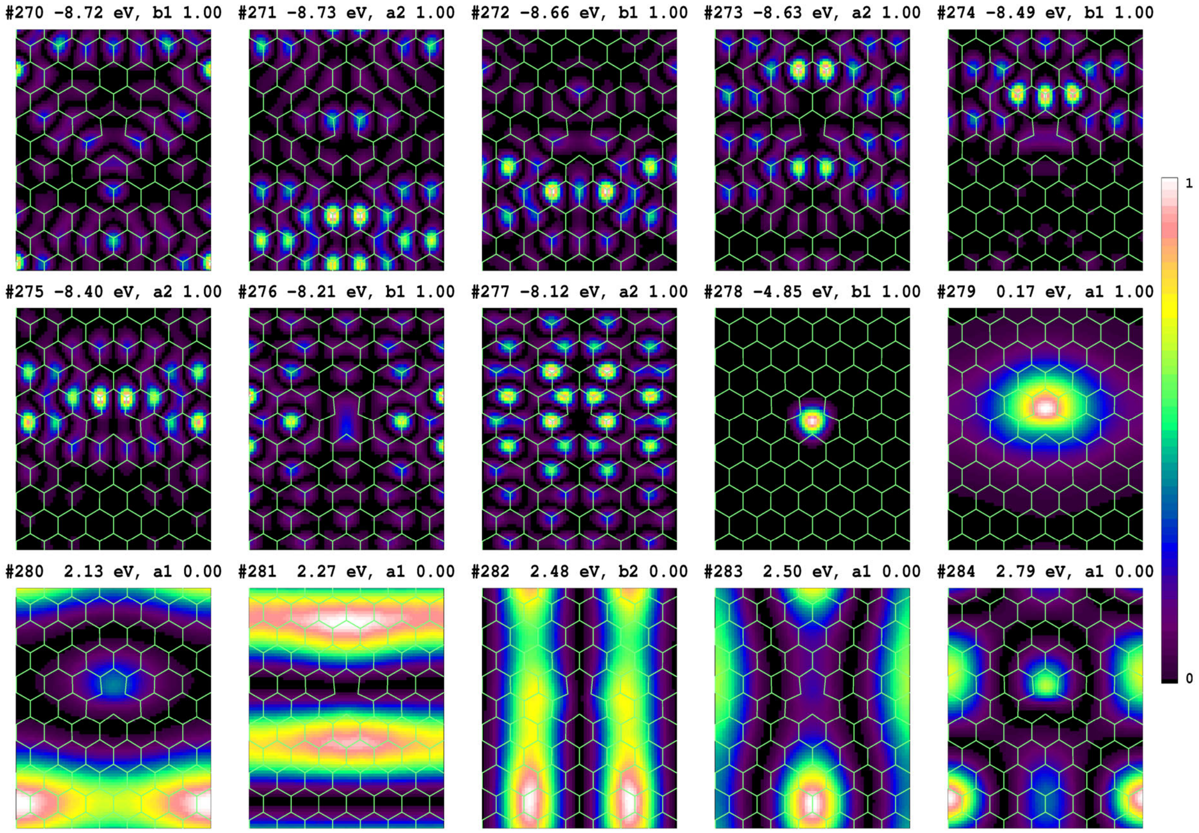

Fig. S12. Frontier orbitals from CAM-B3LYP, $(1)^3B_1$ state, **P**$_{75}$ 1×1×1 model, showing the relative electron density at a distance of 0.80 Å above the h-BN plane.

Cartesian coordinates and calculation details for **1**, **2**, **3**, **4**, $\mathbf{P_{53}}$, $\mathbf{P_{64}}$, and $\mathbf{P_{75}}$ used in the vertical excitation-energy calculations, as well as for the 3D materials optimised by CAM-B3LYP (Tables 3, S1, and S2).

```
1 CAM-B3LYP/6-31G* (1)1A1'
  7    2.316286    0.044168    0.000000
  7   -2.316286    0.044168    0.000000
  7   -1.119892   -2.028046    0.000000
  7    1.196393    1.983878    0.000000
  7   -1.196393    1.983878    0.000000
  7    1.119892   -2.028046    0.000000
  5    0.000000    1.097785    0.000000
  5    0.000000   -2.867163    0.000000
  5   -2.483036    1.433581    0.000000
  5    2.483036    1.433581    0.000000
  5   -0.950709   -0.548892    0.000000
  5    0.950709   -0.548892    0.000000
  1    0.000000   -4.072166    0.000000
  1    1.017486    2.976542    0.000000
  1   -1.017486    2.976542    0.000000
  1   -3.086503   -0.607102    0.000000
  1    3.086503   -0.607102    0.000000
  1   -2.069018   -2.369439    0.000000
  1    2.069018   -2.369439    0.000000
  1   -3.526599    2.036083    0.000000
  1    3.526599    2.036083    0.000000

 #P cam-b3lyp/6-31g* opt guess=read
 Full point group                  D3H     NOp  12
 SCF Done:  E(RCAM-B3LYP) =  -483.103626497     A.U. after    8 cycles
```

```
2 CAM-B3LYP/6-31G* (1)1A1'
  7    6.240389    2.170220    0.000000
  7    4.999660    4.319225    0.000000
  7    3.738983    6.414858    0.000000
  7    3.740000    2.159290    0.000000
  7    2.478065    4.266132    0.000000
  7    1.242707    6.405242    0.000000
  7    1.215847    2.063588    0.000000
  7    0.000000   -4.318580    0.000000
  7   -7.424921    0.030626    0.000000
  7   -6.168456   -2.126405    0.000000
  7   -4.925749   -4.278837    0.000000
  7   -4.933611    0.013002    0.000000
  7   -3.685938   -6.445483    0.000000
  7   -3.689553   -2.130165    0.000000
  7   -2.455546   -4.279134    0.000000
  7   -2.395043    0.021160    0.000000
  7   -1.240729   -6.489445    0.000000
  7   -1.179196   -2.084748    0.000000
  7    7.424921    0.030626    0.000000
  7    6.168456   -2.126405    0.000000
  7    4.925749   -4.278837    0.000000
  7    4.933611    0.013002    0.000000
  7    3.685938   -6.445483    0.000000
  7    3.689553   -2.130165    0.000000
  7    2.455546   -4.279134    0.000000
  7    2.395043    0.021160    0.000000
  7    1.240729   -6.489445    0.000000
```

7	1.179196	-2.084748	0.000000
7	0.000000	4.260329	0.000000
5	0.000000	1.157317	0.000000
7	-6.240389	2.170220	0.000000
7	-4.999660	4.319225	0.000000
7	-3.738983	6.414858	0.000000
7	-3.740000	2.159290	0.000000
7	-2.478065	4.266132	0.000000
7	-1.242707	6.405242	0.000000
7	-1.215847	2.063588	0.000000
5	7.476979	1.442976	0.000000
5	6.236252	3.600502	0.000000
5	4.988143	5.753765	0.000000
5	4.970951	1.443880	0.000000
1	3.730259	7.425017	0.000000
5	3.735912	3.583030	0.000000
5	2.481199	5.705662	0.000000
5	2.478787	1.431128	0.000000
1	1.237997	7.415569	0.000000
5	1.222078	3.501107	0.000000
5	0.000000	-2.862257	0.000000
5	0.000000	-7.201003	0.000000
5	-6.181848	-0.704049	0.000000
5	-4.934468	-2.848916	0.000000
5	-3.700649	-5.001613	0.000000
5	-3.643087	-0.692203	0.000000
5	-2.488835	-7.196742	0.000000
5	-2.421008	-2.808904	0.000000
5	-1.235039	-5.026910	0.000000
5	-1.002266	-0.578659	0.000000
5	6.181848	-0.704049	0.000000
5	4.934468	-2.848916	0.000000
5	3.700649	-5.001613	0.000000
5	3.643087	-0.692203	0.000000
5	2.488835	-7.196742	0.000000
5	2.421008	-2.808904	0.000000
5	1.235039	-5.026910	0.000000
5	1.002266	-0.578659	0.000000
5	0.000000	5.697833	0.000000
5	-7.476979	1.442976	0.000000
5	-6.236252	3.600502	0.000000
5	-4.988143	5.753765	0.000000
5	-4.970951	1.443880	0.000000
1	-3.730259	7.425017	0.000000
5	-3.735912	3.583030	0.000000
5	-2.481199	5.705662	0.000000
5	-2.478787	1.431128	0.000000
1	-1.237997	7.415569	0.000000
5	-1.222078	3.501107	0.000000
1	4.565124	-6.943008	0.000000
1	5.803073	-4.779922	0.000000
1	7.041070	-2.635647	0.000000
1	8.295383	-0.482009	0.000000
1	8.530740	2.013969	0.000000
1	7.273528	4.199373	0.000000
1	6.009518	6.380853	0.000000
1	-6.009518	6.380853	0.000000
1	-7.273528	4.199373	0.000000
1	-8.530740	2.013969	0.000000
1	-8.295383	-0.482009	0.000000
1	-7.041070	-2.635647	0.000000
1	-5.803073	-4.779922	0.000000
1	-4.565124	-6.943008	0.000000

```
  1   -2.521222   -8.394822    0.000000
  1    0.000000   -8.398747    0.000000
  1    2.521222   -8.394822    0.000000

 #P cam-b3lyp/6-31g* opt
 Full point group                 D3H     NOp  12
 SCF Done:  E(RCAM-B3LYP) =  -2880.89254459     A.U. after    6 cycles
```

---

3 CAM-B3LYP/6-31G* (1)1A1'

```
  7    3.756274   -2.168686    0.000000
  7    2.469887   -4.221535    0.000000
  7    1.215972   -2.052769    0.000000
  7    0.000000    4.337372    0.000000
  7   -4.890900   -0.028217    0.000000
  7   -3.651078    2.107951    0.000000
  7   -2.421013    4.249752    0.000000
  7   -2.385736   -0.026678    0.000000
  7   -1.169764    2.079447    0.000000
  7    4.890900   -0.028217    0.000000
  7    3.651078    2.107951    0.000000
  7    2.421013    4.249752    0.000000
  7    2.385736   -0.026678    0.000000
  7    1.169764    2.079447    0.000000
  7    0.000000   -4.215902    0.000000
  7   -3.756274   -2.168686    0.000000
  7   -2.469887   -4.221535    0.000000
  7   -1.215972   -2.052769    0.000000
  5    0.000000   -1.150148    0.000000
  5    4.979489   -1.435417    0.000000
  5    3.732852   -3.594656    0.000000
  5    2.483586   -1.433899    0.000000
  5    1.217969   -3.486490    0.000000
  5    0.000000    2.867798    0.000000
  5   -3.628373    0.688453    0.000000
  5   -2.410404    2.798037    0.000000
  5   -1.246637    5.030073    0.000000
  5   -0.996058    0.575074    0.000000
  5    3.628373    0.688453    0.000000
  5    2.410404    2.798037    0.000000
  5    1.246637    5.030073    0.000000
  5    0.996058    0.575074    0.000000
  5   -4.979489   -1.435417    0.000000
  5   -3.732852   -3.594656    0.000000
  5   -2.483586   -1.433899    0.000000
  5   -1.217969   -3.486490    0.000000
  1    2.427326   -5.230570    0.000000
  1   -5.743469    0.513159    0.000000
  1   -4.525219    2.612637    0.000000
  1   -3.316143    4.717410    0.000000
  1    5.743469    0.513159    0.000000
  1    4.525219    2.612637    0.000000
  1    3.316143    4.717410    0.000000
  1    0.000000   -5.225273    0.000000
  1   -2.427326   -5.230570    0.000000
  1    6.046043   -1.989721    0.000000
  1    4.746170   -4.241166    0.000000
  1   -1.299873    6.230887    0.000000
  1    1.299873    6.230887    0.000000
  1   -6.046043   -1.989721    0.000000
  1   -4.746170   -4.241166    0.000000
```

```
#P cam-b3lyp/6-31G* opt
Full point group                 D3H     NOp  12
SCF Done:  E(RCAM-B3LYP) =  -1442.90595911     A.U. after    9 cycles
```

---

4 CAM-B3LYP/6-31G* (1)1A1'

```
 7    6.231408   -2.163526    0.000000
 7    4.989372   -4.314794    0.000000
 7    3.738188   -6.453073    0.000000
 7    3.737536   -2.157867    0.000000
 7    2.476823   -4.272922    0.000000
 7    1.244464   -6.442661    0.000000
 7    1.215845   -2.066613    0.000000
 7    0.000000    4.315735    0.000000
 7   -7.457619   -0.010829    0.000000
 7   -6.201740    2.143593    0.000000
 7   -4.957276    4.299068    0.000000
 7   -4.938871   -0.008531    0.000000
 7   -3.719431    6.463902    0.000000
 7   -3.696972    2.134448    0.000000
 7   -2.462048    4.281453    0.000000
 7   -2.397662   -0.019647    0.000000
 7   -1.242036    6.478320    0.000000
 7   -1.181816    2.086259    0.000000
 7    7.457619   -0.010829    0.000000
 7    6.201740    2.143593    0.000000
 7    4.957276    4.299068    0.000000
 7    4.938871   -0.008531    0.000000
 7    3.719431    6.463902    0.000000
 7    3.696972    2.134448    0.000000
 7    2.462048    4.281453    0.000000
 7    2.397662   -0.019647    0.000000
 7    1.242036    6.478320    0.000000
 7    1.181816    2.086259    0.000000
 7    0.000000   -4.268896    0.000000
 7   -6.231408   -2.163526    0.000000
 7   -4.989372   -4.314794    0.000000
 7   -3.738188   -6.453073    0.000000
 7   -3.737536   -2.157867    0.000000
 7   -2.476823   -4.272922    0.000000
 7   -1.244464   -6.442661    0.000000
 7   -1.215845   -2.066613    0.000000
 7    8.735864   -2.174189    0.000000
 7    7.492545   -4.325823    0.000000
 7    6.250835   -6.478386    0.000000
 7    0.000000    8.651646    0.000000
 7   -2.485029    8.652575    0.000000
 7    2.485029    8.652575    0.000000
 7   -8.735864   -2.174189    0.000000
 7   -7.492545   -4.325823    0.000000
 7   -6.250835   -6.478386    0.000000
 7    5.001770   -8.590956    0.000000
 7    2.494925   -8.586305    0.000000
 7    0.000000   -8.581669    0.000000
 7   -8.683420    2.132484    0.000000
 7   -9.940871   -0.036182    0.000000
 7   -7.431943    4.290834    0.000000
 7   -6.188495    6.453821    0.000000
 7   -4.939101    8.627138    0.000000
 7    9.940871   -0.036182    0.000000
 7    8.683420    2.132484    0.000000
```

7	7.431943	4.290834	0.000000
7	6.188495	6.453821	0.000000
7	4.939101	8.627138	0.000000
7	-5.001770	-8.590956	0.000000
7	-2.494925	-8.586305	0.000000
5	0.000000	-1.159029	0.000000
5	7.471932	-1.446096	0.000000
5	6.230589	-3.597232	0.000000
5	4.988322	-5.747835	0.000000
5	4.968628	-1.441444	0.000000
5	3.732641	-3.582236	0.000000
5	2.483507	-5.712001	0.000000
5	2.477922	-1.430629	0.000000
5	1.221960	-3.505450	0.000000
5	0.000000	2.861258	0.000000
5	0.000000	7.194465	0.000000
5	-6.188491	0.705220	0.000000
5	-4.940756	2.852547	0.000000
5	-3.704984	5.006781	0.000000
5	-3.646789	0.694477	0.000000
5	-2.483610	7.193931	0.000000
5	-2.424829	2.810973	0.000000
5	-1.235987	5.023680	0.000000
5	-1.003749	0.579515	0.000000
5	6.188491	0.705220	0.000000
5	4.940756	2.852547	0.000000
5	3.704984	5.006781	0.000000
5	3.646789	0.694477	0.000000
5	2.483610	7.193931	0.000000
5	2.424829	2.810973	0.000000
5	1.235987	5.023680	0.000000
5	1.003749	0.579515	0.000000
5	0.000000	-5.705094	0.000000
5	-7.471932	-1.446096	0.000000
5	-6.230589	-3.597232	0.000000
5	-4.988322	-5.747835	0.000000
5	-4.968628	-1.441444	0.000000
5	-3.732641	-3.582236	0.000000
5	-2.483507	-5.712001	0.000000
5	-2.477922	-1.430629	0.000000
5	-1.221960	-3.505450	0.000000
5	3.739994	-7.894800	0.000000
5	1.246050	-7.884710	0.000000
5	-8.707095	0.708470	0.000000
5	-7.451384	2.863244	0.000000
5	-6.205334	5.021466	0.000000
5	-4.967100	7.186330	0.000000
5	8.707095	0.708470	0.000000
5	7.451384	2.863244	0.000000
5	6.205334	5.021466	0.000000
5	4.967100	7.186330	0.000000
5	-3.739994	-7.894800	0.000000
5	-1.246050	-7.884710	0.000000
5	8.732140	-3.605799	0.000000
5	9.978280	-1.450511	0.000000
5	7.488783	-5.759356	0.000000
5	6.245319	-7.916189	0.000000
5	-1.243357	9.365155	0.000000
5	1.243357	9.365155	0.000000
5	-3.732961	9.366699	0.000000
5	3.732961	9.366699	0.000000
5	-9.978280	-1.450511	0.000000
5	-8.732140	-3.605799	0.000000

```
  5   -7.488783   -5.759356    0.000000
  5   -6.245319   -7.916189    0.000000
  1    5.008067   -9.601132    0.000000
  1    2.489629   -9.597049    0.000000
  1    0.000000   -9.592546    0.000000
  1   -9.556102    2.642443    0.000000
  1  -10.818858    0.463453    0.000000
  1   -8.307388    4.796273    0.000000
  1   -7.066474    6.954606    0.000000
  1   -5.810791    9.137679    0.000000
  1   10.818858    0.463453    0.000000
  1    9.556102    2.642443    0.000000
  1    8.307388    4.796273    0.000000
  1    7.066474    6.954606    0.000000
  1    5.810791    9.137679    0.000000
  1   -5.008067   -9.601132    0.000000
  1   -2.489629   -9.597049    0.000000
  1    9.767696   -4.203496    0.000000
  1   11.025961   -2.029696    0.000000
  1    8.524182   -6.357325    0.000000
  1    7.270749   -8.533914    0.000000
  1   -1.243514   10.560821    0.000000
  1    1.243514   10.560821    0.000000
  1   -3.755212   10.563611    0.000000
  1    3.755212   10.563611    0.000000
  1  -11.025961   -2.029696    0.000000
  1   -9.767696   -4.203496    0.000000
  1   -8.524182   -6.357325    0.000000
  1   -7.270749   -8.533914    0.000000

 #P cam-b3lyp/6-31g* opt
 Full point group                 D3H      NOp   6  conv to D3h
 SCF Done:  E(RCAM-B3LYP) =  -4797.06156136     A.U. after    6 cycles
```

P$_{53}$ HSE06 (1)1A1' optimized geometry, wavefunction analysis for (1)3B1

```
  7    0.000000    7.547336    9.913726
  7    0.000000    7.554330    5.553327
  7    0.000000    7.564891    1.203451
  7    0.000000    8.799635   12.097189
  7    0.000000    8.817038    7.742234
  7    0.000000    8.827394    3.371403
  7    0.000000   10.074955    9.956286
  7    0.000000   10.082319    5.544104
  7    0.000000   10.132079    1.192005
  7    0.000000   11.321004    7.762991
  7    0.000000   11.339178   12.183581
  7    0.000000   11.373173    3.334590
  7    0.000000    0.000000    9.962429
  7    0.000000    0.000000    5.577399
  7    0.000000    1.201515    3.334590
  7    0.000000    1.235510   12.183581
  7    0.000000    1.253684    7.762991
  7    0.000000    2.442609    1.192005
  7    0.000000    2.492369    5.544104
  7    0.000000    2.499733    9.956286
  7    0.000000    3.747294    3.371403
  7    0.000000    3.757650    7.742234
  7    0.000000    3.775053   12.097189
  7    0.000000    5.009797    1.203451
```

```
  7    0.000000    5.020358    5.553327
  7    0.000000    5.027352    9.913726
  7    0.000000    6.287344    3.377435
  7    0.000000    6.287344    7.731495
  7    0.000000    6.287344   12.092157
  5    0.000000    7.550901    8.460869
  5    0.000000    7.549731   12.820473
  5    0.000000    7.562470    4.092788
  5    0.000000    8.805696   10.655350
  5    0.000000    8.823368    6.276080
  5    0.000000    8.864643    1.914964
  5    0.000000   10.065827   12.832008
  5    0.000000   10.071721    8.496383
  5    0.000000   10.111116    4.063136
  5    0.000000   11.325149    6.298497
  5    0.000000   11.337281   10.725595
  5    0.000000   11.516458    1.820937
  5    0.000000    0.000000    8.504437
  5    0.000000    0.000000    0.000030
  5    0.000000    0.000000    4.109907
  5    0.000000    1.058230    1.820937
  5    0.000000    1.237407   10.725595
  5    0.000000    1.249539    6.298497
  5    0.000000    2.463572    4.063136
  5    0.000000    2.502967    8.496383
  5    0.000000    2.508861   12.832008
  5    0.000000    3.710045    1.914964
  5    0.000000    3.751320    6.276080
  5    0.000000    3.768992   10.655350
  5    0.000000    5.012218    4.092788
  5    0.000000    5.024957   12.820473
  5    0.000000    5.023787    8.460869
  5    0.000000    6.287344    1.921368
  5    0.000000    6.287344    6.274790
  5    0.000000    6.287344   10.640867

E= -593.1388 eV
box:  15.000000 0 0 0  12.574688 0 0 0  13.068000
Kpoints:  1  1  1
basis: NGX= 112 NGY=  96 NGZ=  96 NGXF= 168 NGYF= 140 NGZF= 144
 total occupation:
 Sym  alpha   beta
 a1   48.00   47.00
 a2   12.00   12.00
 b1   18.00   17.00
 b2   41.00   41.00
 Full wavefunction symmetry=  B1,   NUPDOWN=          2    NOPEN=          2
```

P_{64} HSE06 (1)1A1' optimized geometry, wavefunction analysis for (1)3B1

```
  7    0.000000    0.000000   13.842288
  7    0.000000    0.000000    5.089951
  7    0.000000    0.000000    9.459440
  7    0.000000    7.544814    9.436586
  7    0.000000    7.544814    5.074651
  7    0.000000    7.544814   13.798175
  7    0.000000    7.544814    0.729300
  7    0.000000    8.804752   11.619243
  7    0.000000    6.284875   11.619243
  7    0.000000    8.807645    7.254254
```

7	0.000000	6.281982	7.254254
7	0.000000	8.812816	2.898419
7	0.000000	6.276811	2.898419
7	0.000000	8.802919	15.978926
7	0.000000	6.286708	15.978926
7	0.000000	10.066570	9.441583
7	0.000000	5.023057	9.441583
7	0.000000	10.073186	5.070829
7	0.000000	5.016441	5.070829
7	0.000000	10.061733	13.803887
7	0.000000	5.027894	13.803887
7	0.000000	10.092335	0.727726
7	0.000000	4.997292	0.727726
7	0.000000	11.325291	11.637747
7	0.000000	3.764336	11.637747
7	0.000000	11.327737	7.253224
7	0.000000	3.761890	7.253224
7	0.000000	11.347696	2.890188
7	0.000000	3.741931	2.890188
7	0.000000	11.316347	15.980839
7	0.000000	3.773280	15.980839
7	0.000000	12.578248	9.453397
7	0.000000	2.511379	9.453397
7	0.000000	12.598307	5.057981
7	0.000000	2.491320	5.057981
7	0.000000	12.590277	13.839568
7	0.000000	2.499350	13.839568
7	0.000000	12.654722	0.714053
7	0.000000	2.434905	0.714053
7	0.000000	13.836040	11.646713
7	0.000000	1.253587	11.646713
7	0.000000	13.835157	7.271049
7	0.000000	1.254470	7.271049
7	0.000000	13.890568	2.847898
7	0.000000	1.199059	2.847898
7	0.000000	13.854814	16.062287
7	0.000000	1.234813	16.062287
5	0.000000	0.000000	3.623794
5	0.000000	0.000000	12.390970
5	0.000000	0.000000	8.000084
5	0.000000	7.544814	7.979498
5	0.000000	7.544814	12.347304
5	0.000000	7.544814	3.616864
5	0.000000	7.544814	16.702411
5	0.000000	8.805525	10.165691
5	0.000000	6.284102	10.165691
5	0.000000	8.811192	5.795165
5	0.000000	6.278435	5.795165
5	0.000000	8.804305	14.529332
5	0.000000	6.285322	14.529332
5	0.000000	8.822564	1.445137
5	0.000000	6.267063	1.445137
5	0.000000	10.069526	7.981540
5	0.000000	5.020101	7.981540
5	0.000000	10.063168	12.355765
5	0.000000	5.026459	12.355765
5	0.000000	10.086806	3.611116
5	0.000000	5.002821	3.611116
5	0.000000	10.069952	16.703644
5	0.000000	5.019675	16.703644
5	0.000000	11.323372	10.181779
5	0.000000	3.766255	10.181779
5	0.000000	11.340177	5.786931

```
  5    0.000000    3.749450    5.786931
  5    0.000000   11.322801   14.541710
  5    0.000000    3.766826   14.541710
  5    0.000000   11.390783    1.436086
  5    0.000000    3.698844    1.436086
  5    0.000000   12.580553    7.993712
  5    0.000000    2.509074    7.993712
  5    0.000000   12.584803   12.385216
  5    0.000000    2.504824   12.385216
  5    0.000000   12.630997    3.577241
  5    0.000000    2.458630    3.577241
  5    0.000000   12.583575   16.712245
  5    0.000000    2.506052   16.712245
  5    0.000000   13.840401    5.806471
  5    0.000000    1.249226    5.806471
  5    0.000000   13.834400   10.191118
  5    0.000000    1.255227   10.191118
  5    0.000000   13.853240   14.608376
  5    0.000000    1.236387   14.608376
  5    0.000000   14.038216    1.337572
  5    0.000000    1.051411    1.337572
  5    0.000000    0.000000   16.947396

E= -960.8142 eV
box:  15.000000 0 0 0  15.089627 0 0 0  17.424000
Kpoints:  1  1  1
basis: NGX= 112 NGY= 112 NGZ= 128 NGXF= 168 NGYF= 168 NGZF= 192
 total occupation:
 Sym  alpha   beta
 a1   75.97   75.00
 a2   20.00   20.00
 b1   28.00   27.00
 b2   67.03   67.00
Full wavefunction symmetry=  B1,   NUPDOWN=          2    NOPEN=          2
Projection of occupied orbitals onto (1)1A1 orbitals: 379.23 occupied and
0.95 unoccupied
```

P75 HSE06 (1)1A1' optimized geometry, wavefunction analysis for (1)3B1

```
  7    0.000000    8.802283    2.900712
  7    0.000000    8.802283    7.254790
  7    0.000000    8.802283   11.616811
  7    0.000000    8.802283   15.976529
  7    0.000000    8.802283   20.332494
  7    0.000000   10.070476    0.727334
  7    0.000000   10.064534    5.074672
  7    0.000000   10.062101    9.435313
  7    0.000000   10.061131   13.798657
  7    0.000000   10.059867   18.155769
  7    0.000000   11.320813   20.333239
  7    0.000000   11.320176   15.981302
  7    0.000000   11.321248   11.619805
  7    0.000000   11.323643    7.253393
  7    0.000000   11.333946    2.899068
  7    0.000000   12.577738   18.161003
  7    0.000000   12.580745    9.436462
  7    0.000000   12.579761   13.807524
  7    0.000000   12.589348    5.071031
  7    0.000000   12.610506    0.726246
  7    0.000000   13.833768   20.337028
```

7	0.000000	13.835361	11.627216
7	0.000000	13.841233	15.998507
7	0.000000	13.842909	7.249978
7	0.000000	13.864482	2.888553
7	0.000000	15.091857	9.446296
7	0.000000	15.093060	13.816026
7	0.000000	15.106578	18.198328
7	0.000000	15.113436	5.055228
7	0.000000	15.170818	0.713126
7	0.000000	16.348319	11.633654
7	0.000000	16.349528	7.264566
7	0.000000	16.351102	16.006171
7	0.000000	16.373210	20.419990
7	0.000000	16.405378	2.846399
7	0.000000	0.000000	9.449327
7	0.000000	0.000000	5.086013
7	0.000000	0.000000	13.818957
7	0.000000	0.000000	18.200516
7	0.000000	1.199187	2.846399
7	0.000000	1.231355	20.419990
7	0.000000	1.253463	16.006171
7	0.000000	1.255037	7.264566
7	0.000000	1.256246	11.633654
7	0.000000	2.433747	0.713126
7	0.000000	2.491129	5.055228
7	0.000000	2.497987	18.198328
7	0.000000	2.512708	9.446296
7	0.000000	2.511505	13.816026
7	0.000000	3.740083	2.888553
7	0.000000	3.761656	7.249978
7	0.000000	3.763332	15.998507
7	0.000000	3.769204	11.627216
7	0.000000	3.770797	20.337028
7	0.000000	4.994059	0.726246
7	0.000000	5.015217	5.071031
7	0.000000	5.023820	9.436462
7	0.000000	5.024804	13.807524
7	0.000000	5.026827	18.161003
7	0.000000	6.270619	2.899068
7	0.000000	6.280922	7.253393
7	0.000000	6.283317	11.619805
7	0.000000	6.284389	15.981302
7	0.000000	6.283752	20.333239
7	0.000000	7.534089	0.727334
7	0.000000	7.542464	9.435313
7	0.000000	7.540031	5.074672
7	0.000000	7.543434	13.798657
7	0.000000	7.544698	18.155769
5	0.000000	8.802283	1.447031
5	0.000000	8.802283	5.797259
5	0.000000	8.802283	10.161482
5	0.000000	8.802283	14.526282
5	0.000000	8.802283	18.882835
5	0.000000	10.070158	3.618648
5	0.000000	10.063868	7.978402
5	0.000000	10.061597	12.345613
5	0.000000	10.061159	16.706957
5	0.000000	10.065120	21.056768
5	0.000000	11.321567	18.885529
5	0.000000	11.322303	10.163010
5	0.000000	11.320006	14.531194
5	0.000000	11.329939	5.794601
5	0.000000	11.346758	1.445509

```
  5    0.000000   12.579512   16.715976
  5    0.000000   12.578585   12.353688
  5    0.000000   12.585510    7.976406
  5    0.000000   12.588505   21.059124
  5    0.000000   12.606943    3.611446
  5    0.000000   13.839413   18.899253
  5    0.000000   13.836080   10.170595
  5    0.000000   13.838025   14.546292
  5    0.000000   13.856764    5.783920
  5    0.000000   13.908919    1.435067
  5    0.000000   15.092052   12.361924
  5    0.000000   15.095660    7.985850
  5    0.000000   15.101287   21.068507
  5    0.000000   15.101036   16.746519
  5    0.000000   15.147243    3.574854
  5    0.000000   16.348631   10.176339
  5    0.000000   16.349409   14.554222
  5    0.000000   16.355674    5.800630
  5    0.000000   16.369505   18.967920
  5    0.000000   16.554415    1.337054
  5    0.000000    0.000000    7.989343
  5    0.000000    0.000000    3.621007
  5    0.000000    0.000000   12.365043
  5    0.000000    0.000000   16.751958
  5    0.000000    0.000000   21.306485
  5    0.000000    1.050150    1.337054
  5    0.000000    1.235060   18.967920
  5    0.000000    1.248891    5.800630
  5    0.000000    1.255934   10.176339
  5    0.000000    1.255156   14.554222
  5    0.000000    2.457322    3.574854
  5    0.000000    2.503529   16.746519
  5    0.000000    2.503278   21.068507
  5    0.000000    2.508905    7.985850
  5    0.000000    2.512513   12.361924
  5    0.000000    3.695646    1.435067
  5    0.000000    3.747801    5.783920
  5    0.000000    3.766540   14.546292
  5    0.000000    3.768485   10.170595
  5    0.000000    3.765152   18.899253
  5    0.000000    4.997622    3.611446
  5    0.000000    5.016060   21.059124
  5    0.000000    5.019055    7.976406
  5    0.000000    5.025980   12.353688
  5    0.000000    5.025053   16.715976
  5    0.000000    6.257807    1.445509
  5    0.000000    6.274626    5.794601
  5    0.000000    6.284559   14.531194
  5    0.000000    6.282262   10.163010
  5    0.000000    6.282998   18.885529
  5    0.000000    7.534407    3.618648
  5    0.000000    7.542968   12.345613
  5    0.000000    7.540697    7.978402
  5    0.000000    7.543406   16.706957
  5    0.000000    7.539445   21.056768

E=-1410.1872 eV
box:  15.000000 0 0 0  17.604565 0 0 0  21.780001
Kpoints:  1  1  1
basis: NGX= 112 NGY= 128 NGZ= 160 NGXF= 168 NGYF= 192 NGZF= 240
 total occupation:
 Sym  alpha   beta
 a1  109.84  109.00
```

```
a2   30.00   30.00
b1   40.00   39.00
b2   99.16   99.00
Full wavefunction symmetry=  B1,   NUPDOWN=          2    NOPEN=          2
```

P_{53} CAM-B3LYP (1)1A1' optimized geometry, wavefunction analysis for (1)3B1

```
7    0.000000    7.547336    9.913726
7    0.000000    7.554330    5.553327
7    0.000000    7.564891    1.203451
7    0.000000    8.799635   12.097189
7    0.000000    8.817038    7.742234
7    0.000000    8.827394    3.371403
7    0.000000   10.074955    9.956286
7    0.000000   10.082319    5.544104
7    0.000000   10.132079    1.192005
7    0.000000   11.321004    7.762991
7    0.000000   11.339178   12.183581
7    0.000000   11.373173    3.334590
7    0.000000    0.000000    9.962429
7    0.000000    0.000000    5.577399
7    0.000000    1.201515    3.334590
7    0.000000    1.235510   12.183581
7    0.000000    1.253684    7.762991
7    0.000000    2.442609    1.192005
7    0.000000    2.492369    5.544104
7    0.000000    2.499733    9.956286
7    0.000000    3.747294    3.371403
7    0.000000    3.757650    7.742234
7    0.000000    3.775053   12.097189
7    0.000000    5.009797    1.203451
7    0.000000    5.020358    5.553327
7    0.000000    5.027352    9.913726
7    0.000000    6.287344    3.377435
7    0.000000    6.287344    7.731495
7    0.000000    6.287344   12.092157
5    0.000000    7.550901    8.460869
5    0.000000    7.549731   12.820473
5    0.000000    7.562470    4.092788
5    0.000000    8.805696   10.655350
5    0.000000    8.823368    6.276080
5    0.000000    8.864643    1.914964
5    0.000000   10.065827   12.832008
5    0.000000   10.071721    8.496383
5    0.000000   10.111116    4.063136
5    0.000000   11.325149    6.298497
5    0.000000   11.337281   10.725595
5    0.000000   11.516458    1.820937
5    0.000000    0.000000    8.504437
5    0.000000    0.000000    0.000030
5    0.000000    0.000000    4.109907
5    0.000000    1.058230    1.820937
5    0.000000    1.237407   10.725595
5    0.000000    1.249539    6.298497
5    0.000000    2.463572    4.063136
5    0.000000    2.502967    8.496383
5    0.000000    2.508861   12.832008
5    0.000000    3.710045    1.914964
5    0.000000    3.751320    6.276080
5    0.000000    3.768992   10.655350
5    0.000000    5.012218    4.092788
```

```
  5    0.000000    5.024957   12.820473
  5    0.000000    5.023787    8.460869
  5    0.000000    6.287344    1.921368
  5    0.000000    6.287344    6.274790
  5    0.000000    6.287344   10.640867

E=-1012.0805 eV
box:  15.000000 0 0 0  12.574688 0 0 0  13.068000
Kpoints:  1  1  1
basis: NGX=  112 NGY=  96 NGZ=  98 NGXF= 168 NGYF= 192 NGZF= 196
 Sym  alpha   beta
 a1   48.00   47.00
 a2   12.00   12.00
 b1   18.00   17.00
 b2   41.00   41.00
 Full wavefunction symmetry=  B1,   NUPDOWN=          2    NOPEN=          2
```

P_{64} CAM-B3LYP (1)1A1' optimized geometry, wavefunction analysis for (1)3B1

```
  7    0.000000    0.000000    5.067367
  7    0.000000    0.000000   13.610362
  7    0.000000    0.000000    0.737847
  7    0.000000    7.442601    0.713036
  7    0.000000    7.442601   13.597526
  7    0.000000    7.442601    5.018241
  7    0.000000    7.442601    9.313487
  7    0.000000    8.685447    2.868289
  7    0.000000    6.199755    2.868289
  7    0.000000    8.689360   15.747125
  7    0.000000    6.195841   15.747125
  7    0.000000    8.695209   11.453341
  7    0.000000    6.189993   11.453341
  7    0.000000    8.684592    7.170239
  7    0.000000    6.200609    7.170239
  7    0.000000    9.930702    0.719913
  7    0.000000    4.954500    0.719913
  7    0.000000    9.938112   13.594680
  7    0.000000    4.947090   13.594680
  7    0.000000    9.926879    5.022819
  7    0.000000    4.958323    5.022819
  7    0.000000    9.957331    9.312150
  7    0.000000    4.927871    9.312150
  7    0.000000   11.172460    2.886578
  7    0.000000    3.712742    2.886578
  7    0.000000   11.175910   15.746987
  7    0.000000    3.709292   15.746987
  7    0.000000   11.197361   11.443337
  7    0.000000    3.687840   11.443337
  7    0.000000   11.166729    7.172949
  7    0.000000    3.718473    7.172949
  7    0.000000   12.408749    0.732270
  7    0.000000    2.476453    0.732270
  7    0.000000   12.428711   13.579247
  7    0.000000    2.456491   13.579247
  7    0.000000   12.421876    5.061343
  7    0.000000    2.463325    5.061343
  7    0.000000   12.492720    9.294466
  7    0.000000    2.392482    9.294466
  7    0.000000   13.649031    2.897440
  7    0.000000    1.236170    2.897440
  7    0.000000   13.645774   15.764055
```

```
  7    0.000000    1.239428   15.764055
  7    0.000000   13.712851   11.399215
  7    0.000000    1.172351   11.399215
  7    0.000000   13.667596    7.262775
  7    0.000000    1.217606    7.262775
  5    0.000000    0.000000   12.159891
  5    0.000000    0.000000    3.624391
  5    0.000000    0.000000   16.479931
  5    0.000000    7.442601   16.462935
  5    0.000000    7.442601    3.585986
  5    0.000000    7.442601   12.159157
  5    0.000000    7.442601    7.878166
  5    0.000000    8.686112    1.433557
  5    0.000000    6.199090    1.433557
  5    0.000000    8.691288   14.309032
  5    0.000000    6.193914   14.309032
  5    0.000000    8.685202    5.737292
  5    0.000000    6.200000    5.737292
  5    0.000000    8.702511   10.015240
  5    0.000000    6.182691   10.015240
  5    0.000000    9.933173   16.464434
  5    0.000000    4.952029   16.464434
  5    0.000000    9.926573    3.593847
  5    0.000000    4.958629    3.593847
  5    0.000000    9.947602   12.154286
  5    0.000000    4.937600   12.154286
  5    0.000000    9.933331    7.878842
  5    0.000000    4.951871    7.878842
  5    0.000000   11.170492    1.449041
  5    0.000000    3.714710    1.449041
  5    0.000000   11.185893   14.299932
  5    0.000000    3.699309   14.299932
  5    0.000000   11.167996    5.748900
  5    0.000000    3.717206    5.748900
  5    0.000000   11.241200   10.006724
  5    0.000000    3.644002   10.006724
  5    0.000000   12.410909   16.475484
  5    0.000000    2.474293   16.475484
  5    0.000000   12.413937    3.621466
  5    0.000000    2.471265    3.621466
  5    0.000000   12.460428   12.115053
  5    0.000000    2.424774   12.115053
  5    0.000000   12.418877    7.889667
  5    0.000000    2.466325    7.889667
  5    0.000000   13.653805   14.318055
  5    0.000000    1.231397   14.318055
  5    0.000000   13.647280    1.456008
  5    0.000000    1.237922    1.456008
  5    0.000000   13.668545    5.819054
  5    0.000000    1.216657    5.819054
  5    0.000000   13.867324    9.900457
  5    0.000000    1.017878    9.900457
  5    0.000000    0.000000    8.144234

E=-1968.5105 eV
box:  15.000000 0 0 0  14.885202 0 0 0  17.185348
Kpoints:  1  1  1
basis: NGX= 112 NGY= 112 NGZ= 126 NGXF= 168 NGYF= 162 NGZF= 192
 Sym  alpha   beta
 a1   76.00   75.00
 a2   20.00   20.00
 b1   28.00   27.00
 b2   67.00   67.00
```

Full wavefunction symmetry= B1,   NUPDOWN=   2   NOPEN=   2

P_{75} CAM-B3LYP (1)1A1' optimized geometry, wavefunction analysis for (1)3B1

7	0.000000	8.802283	2.900712
7	0.000000	8.802283	7.254790
7	0.000000	8.802283	11.616811
7	0.000000	8.802283	15.976529
7	0.000000	8.802283	20.332494
7	0.000000	10.070476	0.727334
7	0.000000	10.064534	5.074672
7	0.000000	10.062101	9.435313
7	0.000000	10.061131	13.798657
7	0.000000	10.059867	18.155769
7	0.000000	11.320813	20.333239
7	0.000000	11.320176	15.981302
7	0.000000	11.321248	11.619805
7	0.000000	11.323643	7.253393
7	0.000000	11.333946	2.899068
7	0.000000	12.577738	18.161003
7	0.000000	12.580745	9.436462
7	0.000000	12.579761	13.807524
7	0.000000	12.589348	5.071031
7	0.000000	12.610506	0.726246
7	0.000000	13.833768	20.337028
7	0.000000	13.835361	11.627216
7	0.000000	13.841233	15.998507
7	0.000000	13.842909	7.249978
7	0.000000	13.864482	2.888553
7	0.000000	15.091857	9.446296
7	0.000000	15.093060	13.816026
7	0.000000	15.106578	18.198328
7	0.000000	15.113436	5.055228
7	0.000000	15.170818	0.713126
7	0.000000	16.348319	11.633654
7	0.000000	16.349528	7.264566
7	0.000000	16.351102	16.006171
7	0.000000	16.373210	20.419990
7	0.000000	16.405378	2.846399
7	0.000000	0.000000	9.449327
7	0.000000	0.000000	5.086013
7	0.000000	0.000000	13.818957
7	0.000000	0.000000	18.200516
7	0.000000	1.199187	2.846399
7	0.000000	1.231355	20.419990
7	0.000000	1.253463	16.006171
7	0.000000	1.255037	7.264566
7	0.000000	1.256246	11.633654
7	0.000000	2.433747	0.713126
7	0.000000	2.491129	5.055228
7	0.000000	2.497987	18.198328
7	0.000000	2.512708	9.446296
7	0.000000	2.511505	13.816026
7	0.000000	3.740083	2.888553
7	0.000000	3.761656	7.249978
7	0.000000	3.763332	15.998507
7	0.000000	3.769204	11.627216
7	0.000000	3.770797	20.337028
7	0.000000	4.994059	0.726246
7	0.000000	5.015217	5.071031
7	0.000000	5.023820	9.436462

7	0.000000	5.024804	13.807524
7	0.000000	5.026827	18.161003
7	0.000000	6.270619	2.899068
7	0.000000	6.280922	7.253393
7	0.000000	6.283317	11.619805
7	0.000000	6.284389	15.981302
7	0.000000	6.283752	20.333239
7	0.000000	7.534089	0.727334
7	0.000000	7.542464	9.435313
7	0.000000	7.540031	5.074672
7	0.000000	7.543434	13.798657
7	0.000000	7.544698	18.155769
5	0.000000	8.802283	1.447031
5	0.000000	8.802283	5.797259
5	0.000000	8.802283	10.161482
5	0.000000	8.802283	14.526282
5	0.000000	8.802283	18.882835
5	0.000000	10.070158	3.618648
5	0.000000	10.063868	7.978402
5	0.000000	10.061597	12.345613
5	0.000000	10.061159	16.706957
5	0.000000	10.065120	21.056768
5	0.000000	11.321567	18.885529
5	0.000000	11.322303	10.163010
5	0.000000	11.320006	14.531194
5	0.000000	11.329939	5.794601
5	0.000000	11.346758	1.445509
5	0.000000	12.579512	16.715976
5	0.000000	12.578585	12.353688
5	0.000000	12.585510	7.976406
5	0.000000	12.588505	21.059124
5	0.000000	12.606943	3.611446
5	0.000000	13.839413	18.899253
5	0.000000	13.836080	10.170595
5	0.000000	13.838025	14.546292
5	0.000000	13.856764	5.783920
5	0.000000	13.908919	1.435067
5	0.000000	15.092052	12.361924
5	0.000000	15.095660	7.985850
5	0.000000	15.101287	21.068507
5	0.000000	15.101036	16.746519
5	0.000000	15.147243	3.574854
5	0.000000	16.348631	10.176339
5	0.000000	16.349409	14.554222
5	0.000000	16.355674	5.800630
5	0.000000	16.369505	18.967920
5	0.000000	16.554415	1.337054
5	0.000000	0.000000	7.989343
5	0.000000	0.000000	3.621007
5	0.000000	0.000000	12.365043
5	0.000000	0.000000	16.751958
5	0.000000	0.000000	21.306485
5	0.000000	1.050150	1.337054
5	0.000000	1.235060	18.967920
5	0.000000	1.248891	5.800630
5	0.000000	1.255934	10.176339
5	0.000000	1.255156	14.554222
5	0.000000	2.457322	3.574854
5	0.000000	2.503529	16.746519
5	0.000000	2.503278	21.068507
5	0.000000	2.508905	7.985850
5	0.000000	2.512513	12.361924
5	0.000000	3.695646	1.435067

```
  5    0.000000    3.747801    5.783920
  5    0.000000    3.766540   14.546292
  5    0.000000    3.768485   10.170595
  5    0.000000    3.765152   18.899253
  5    0.000000    4.997622    3.611446
  5    0.000000    5.016060   21.059124
  5    0.000000    5.019055    7.976406
  5    0.000000    5.025980   12.353688
  5    0.000000    5.025053   16.715976
  5    0.000000    6.257807    1.445509
  5    0.000000    6.274626    5.794601
  5    0.000000    6.284559   14.531194
  5    0.000000    6.282262   10.163010
  5    0.000000    6.282998   18.885529
  5    0.000000    7.534407    3.618648
  5    0.000000    7.542968   12.345613
  5    0.000000    7.540697    7.978402
  5    0.000000    7.543406   16.706957
  5    0.000000    7.539445   21.056768

E=-2633.9578 eV
box:  15.000000 0 0 0  17.604565 0 0 0  21.780001
Kpoints:  1  1  1
basis: NGX=  112 NGY= 128 NGZ= 160 NGXF= 168 NGYF= 192 NGZF= 240
 Sym  alpha   beta
 a1  110.00  109.00
 a2   30.00   30.00
 b1   40.00   39.00
 b2   99.00   99.00
 Full wavefunction symmetry=  B1,   NUPDOWN=          2    NOPEN=           2
```

```
   Al2O3  CAM-B3LYP
 13    0.000000    0.000000    1.922137
 13    0.000000    2.749269    0.244134
 13    0.000000    2.749269    4.088408
 13    2.380937    1.374635    2.410405
 13    2.380937    1.374635    6.254679
 13    0.000000    0.000000    4.576676
 13    0.000000    0.000000    8.420950
 13    0.000000    2.749269    6.742947
 13    0.000000    2.749269   10.587221
 13    2.380937    1.374635    8.909218
 13    2.380937    1.374635   12.753492
 13    0.000000    0.000000   11.075489
  8    3.302766    0.000000    3.249406
  8    1.651383    0.111010    1.083135
  8    0.729554    1.263625    3.249406
  8   -1.651383    2.860279    3.249406
  8    1.651383    2.638260    1.083135
  8    3.840045    1.374635    1.083135
  8    0.921829    1.374635    7.581948
  8   -0.729554    1.485645    5.415677
  8    3.110491    2.638260    7.581948
  8    3.110491    0.111010    7.581948
  8   -0.729554    4.012894    5.415677
  8    1.459108    2.749269    5.415677
  8   -1.459108    2.749269   11.914490
  8    1.651383    2.860279    9.748219
  8    0.729554    4.012894   11.914490
  8    0.729554    1.485645   11.914490
  8   -0.729554    1.263625    9.748219
```

```
  8    1.459108    0.000000    9.748219

E= -307.2240 eV
box:   4.761874 0 0  -2.380937   4.123904 0 0 0  12.997626
Kpoints:  6  6  4
basis: NGX=  36 NGY=  36 NGZ=  48 NGXF=  72 NGYF=  72 NGZF=  96
```

```
   AlAs  CAM-B3LYP
 13    0.000000    0.000000    0.000000
 13    0.000000    2.831784    2.831784
 13    2.831784    0.000000    2.831784
 13    2.831784    2.831784    0.000000
 33    4.247675    1.415892    4.247675
 33    4.247675    4.247675    1.415892
 33    1.415892    1.415892    1.415892
 33    1.415892    4.247675    4.247675

E=  -54.8389 eV
box:   5.663567 0 0 0   5.663567 0 0 0   5.663567
Kpoints:  6  6  6
basis: NGX=  26 NGY=  26 NGZ=  26 NGXF=  52 NGYF=  52 NGZF=  52
```

```
   AlN-wz  CAM-B3LYP
 13    0.000000    1.789336    4.972004
 13    1.549611    0.894668    2.484712
  7    0.000000    1.789336    1.892919
  7    1.549611    0.894668    4.380211

E=  -42.2159 eV
box:   3.099221 0 0  -1.549611   2.684004 0 0 0   4.974584
Kpoints:  8  8  4
basis: NGX=  24 NGY=  24 NGZ=  36 NGXF=  48 NGYF=  48 NGZF=  72
```

```
   AlP-zb  CAM-B3LYP
 13    0.000000    0.000000    0.000000
 13    0.000000    2.729276    2.729276
 13    2.729276    0.000000    2.729276
 13    2.729276    2.729276    0.000000
 15    1.364638    4.093914    4.093914
 15    1.364638    1.364638    1.364638
 15    4.093914    4.093914    1.364638
 15    4.093914    1.364638    4.093914

E=  -54.4228 eV
box:   5.458552 0 0 0   5.458552 0 0 0   5.458552
Kpoints:  8  8  8
basis: NGX=  24 NGY=  24 NGZ=  24 NGXF=  48 NGYF=  48 NGZF=  48
```

```
   BP  CAM-B3LYP
  5    0.000000    0.000000    0.000000
  5    0.000000    2.269041    2.269041
  5    2.269041    0.000000    2.269041
  5    2.269041    2.269041    0.000000
 15    1.134521    1.134521    3.403562
 15    1.134521    3.403562    1.134521
```

```
 15    3.403562    1.134521    1.134521
 15    3.403562    3.403562    3.403562

E=  -16.8536 eV
box:   4.538083 0 0 0   4.538083 0 0 0   4.538083
Kpoints: 10 10 10
basis: NGX=  24 NGY=  24 NGZ=  24 NGXF=  48 NGYF=  48 NGZF=  48
```

```
  CaO  CAM-B3LYP
 20    0.000000    0.000000    0.000000
 20    0.000000    2.406595    2.406595
 20    2.406595    0.000000    2.406595
 20    2.406595    2.406595    0.000000
  8    0.000000    0.000000    2.406595
  8    0.000000    2.406595    0.000000
  8    2.406595    0.000000    0.000000
  8    2.406595    2.406595    2.406595

E=  -65.8148 eV
box:   4.813190 0 0 0   4.813190 0 0 0   4.813190
Kpoints: 10 10 10
basis: NGX=  40 NGY=  40 NGZ=  40 NGXF=  80 NGYF=  80 NGZF=  80
```

```
  GaAs  CAM-B3LYP
 31    0.000000    0.000000    0.000000
 31    0.000000    2.828788    2.828788
 31    2.828788    0.000000    2.828788
 31    2.828788    2.828788    0.000000
 33    1.414394    4.243182    4.243182
 33    1.414394    1.414394    1.414394
 33    4.243182    4.243182    1.414394
 33    4.243182    1.414394    4.243182

E=  -50.7180 eV
box:   5.657575 0 0 0   5.657575 0 0 0   5.657575
Kpoints:  8  8  8
basis: NGX=  32 NGY=  32 NGZ=  32 NGXF=  64 NGYF=  64 NGZF=  64
```

```
  GaN-zb  CAM-B3LYP
 31    0.000000    0.000000    0.000000
 31    0.000000    2.252824    2.252824
 31    2.252824    0.000000    2.252824
 31    2.252824    2.252824    0.000000
  7    1.126412    3.379236    3.379236
  7    1.126412    1.126412    1.126412
  7    3.379236    3.379236    1.126412
  7    3.379236    1.126412    3.379236

E=  -64.5796 eV
box:   4.505647 0 0 0   4.505647 0 0 0   4.505647
Kpoints: 10 10 10
basis: NGX=  28 NGY=  28 NGZ=  28 NGXF=  56 NGYF=  56 NGZF=  56
```

```
  GaP-zb  CAM-B3LYP
 31    0.000000    0.000000    0.000000
```

```
 31    0.000000    2.723626    2.723626
 31    2.723626    0.000000    2.723626
 31    2.723626    2.723626    0.000000
 15    1.361813    4.085438    4.085438
 15    1.361813    1.361813    1.361813
 15    4.085438    4.085438    1.361813
 15    4.085438    1.361813    4.085438

E=  -54.7896 eV
box:   5.447251 0 0 0   5.447251 0 0 0   5.447251
Kpoints:  8  8  8
basis: NGX=  28 NGY=  28 NGZ=  28 NGXF=  56 NGYF=  56 NGZF=  56
```

```
  Ge  CAM-B3LYP
 32    0.000000    0.000000    2.830095
 32    1.415047    1.415047    4.245142
 32    0.000000    2.830095    0.000000
 32    1.415047    4.245142    1.415047
 32    2.830095    0.000000    0.000000
 32    4.245142    1.415047    1.415047
 32    2.830095    2.830095    2.830095
 32    4.245142    4.245142    4.245142

E=  -54.7832 eV
box:   5.660190 0 0 0   5.660190 0 0 0   5.660190
Kpoints:  8  8  8
basis: NGX=  36 NGY=  36 NGZ=  36 NGXF=  72 NGYF=  72 NGZF=  72
```

```
  InAs  CAM-B3LYP
 49    1.515645    4.546936    4.546936
 49    1.515645    1.515645    1.515645
 49    4.546936    4.546936    1.515645
 49    4.546936    1.515645    4.546936
 33    0.000000    0.000000    0.000000
 33    0.000000    3.031291    3.031291
 33    3.031291    0.000000    3.031291
 33    3.031291    3.031291    0.000000

E=  -48.5320 eV
box:   6.062582 0 0 0   6.062582 0 0 0   6.062582
Kpoints: 10 10 10
basis: NGX=  32 NGY=  32 NGZ=  32 NGXF=  64 NGYF=  64 NGZF=  64
```

```
  InP  CAM-B3LYP
 49    0.000000    0.000000    0.000000
 49    0.000000    2.937435    2.937435
 49    2.937435    0.000000    2.937435
 49    2.937435    2.937435    0.000000
 15    1.468718    4.406153    4.406153
 15    1.468718    1.468718    1.468718
 15    4.406153    4.406153    1.468718
 15    4.406153    1.468718    4.406153

E=  -52.6509 eV
box:   5.874871 0 0 0   5.874871 0 0 0   5.874871
Kpoints:  6  6  6
basis: NGX=  26 NGY=  26 NGZ=  26 NGXF=  52 NGYF=  52 NGZF=  52
```

```
  LiCl  CAM-B3LYP
  3    0.000000    0.000000    0.000000
  3    0.000000    2.533431    2.533431
  3    2.533431    0.000000    2.533431
  3    2.533431    2.533431    0.000000
 17    0.000000    0.000000    2.533431
 17    0.000000    2.533431    0.000000
 17    2.533431    0.000000    0.000000
 17    2.533431    2.533431    2.533431

E=  -43.1803 eV
box:   5.066862 0 0 0   5.066862 0 0 0   5.066862
Kpoints:  6  6  6
basis: NGX=  28 NGY=  28 NGZ=  28 NGXF=  56 NGYF=  56 NGZF=  56
```

```
  LiF  CAM-B3LYP
  3    0.000000    0.000000    0.000000
  3    0.000000    1.983998    1.983998
  3    1.983998    0.000000    1.983998
  3    1.983998    1.983998    0.000000
  9    0.000000    0.000000    1.983998
  9    0.000000    1.983998    0.000000
  9    1.983998    0.000000    0.000000
  9    1.983998    1.983998    1.983998

E=  -55.4773 eV
box:   3.967996 0 0 0   3.967996 0 0 0   3.967996
Kpoints:  8  8  8
basis: NGX=  28 NGY=  28 NGZ=  28 NGXF=  56 NGYF=  56 NGZF=  56
```

```
  SiC  CAM-B3LYP
 14    0.000000    2.173588    2.173588
 14    0.000000    0.000000    0.000000
 14    2.173588    2.173588    0.000000
 14    2.173588    0.000000    2.173588
  6    3.260382    3.260382    3.260382
  6    3.260382    1.086794    1.086794
  6    1.086794    3.260382    1.086794
  6    1.086794    1.086794    3.260382

E=  -84.7238 eV
box:   4.347176 0 0 0   4.347176 0 0 0   4.347176
Kpoints:  6  6  6
basis: NGX=  32 NGY=  32 NGZ=  32 NGXF=  66 NGYF=  66 NGZF=  66
```

```
  SiO2  CAM-B3LYP
 14    2.568117    0.000000    0.911771
 14   -1.284059    2.224055    4.558855
 14    1.170619    2.027572    2.735313
  8   -0.804377    3.558239    5.273288
  8    1.029036    3.169118    1.626204
  8   -0.224659    1.775897    3.449746
  8    1.650301    0.693388    2.020880
  8    2.230019    2.475730    3.844422
```

```
  8    3.483714    1.082509    0.197338

E= -108.8484 eV
box:   4.909356 0 0  -2.454678   4.251627 0 0 0   5.470626
Kpoints:  9  9  5
basis: NGX=  30 NGY=  30 NGZ=  32 NGXF=  60 NGYF=  60 NGZF=  64
```

```
 Si B3LYP
 14    1.368089    1.368089    4.104267
 14    0.000000    0.000000    2.736178
 14    1.368089    4.104267    1.368089
 14    0.000000    2.736178    0.000000
 14    4.104267    1.368089    1.368089
 14    2.736178    0.000000    0.000000
 14    4.104267    4.104267    4.104267
 14    2.736178    2.736178    2.736178

E=  -47.3008 eV
box:   5.472356 0 0 0   5.472356 0 0 0   5.472356
Kpoints: 12 12 12
basis: NGX=  24 NGY=  24 NGZ=  24 NGXF=  48 NGYF=  48 NGZF=  48
```

```
 NaCl B3LYP
 11    0.000000    0.000000    0.000000
 11    0.000000    2.835111    2.835111
 11    2.835111    0.000000    2.835111
 11    2.835111    2.835111    0.000000
 17    0.000000    0.000000    2.835111
 17    0.000000    2.835111    0.000000
 17    2.835111    0.000000    0.000000
 17    2.835111    2.835111    2.835111

E=  -31.1417 eV
box:   5.670222 0 0 0   5.670222 0 0 0   5.670222
Kpoints: 12 12 12
basis: NGX=  24 NGY=  24 NGZ=  24 NGXF=  48 NGYF=  48 NGZF=  48
```

```
 MgO B3LYP
 12    0.000000    0.000000    0.000000
 12    0.000000    2.103380    2.103380
 12    2.103380    0.000000    2.103380
 12    2.103380    2.103380    0.000000
  8    0.000000    0.000000    2.103380
  8    0.000000    2.103380    0.000000
  8    2.103380    0.000000    0.000000
  8    2.103380    2.103380    2.103380

E=  -55.3269 eV
box:   4.206761 0 0 0   4.206761 0 0 0   4.206761
Kpoints: 12 12 12
basis: NGX=  24 NGY=  24 NGZ=  24 NGXF=  48 NGYF=  48 NGZF=  48
```

```
 h-BN B3LYP
  7    1.246446    1.439280    4.947575
  7    0.000000    0.719628    1.649192
```

```
  7    0.000000    3.598189    4.947575
  7    1.246446    2.878537    1.649192
  5    0.000000    0.719627    4.947575
  5    1.246446    1.439281    1.649192
  5    1.246446    2.878536    4.947575
  5    0.000000    3.598190    1.649192

E=  -40.1372 eV
box:   2.492893 0 0 0   4.317817 0 0 0   6.596766
Kpoints: 12 12  4
basis: NGX=  24 NGY=  24 NGZ=  64 NGXF=  48 NGYF=  48 NGZF= 128
```

```
 diamond B3LYP
  6    0.000000    0.000000    0.000000
  6    0.889814    2.669441    2.669441
  6    0.000000    1.779627    1.779627
  6    0.889814    0.889814    0.889814
  6    1.779627    0.000000    1.779627
  6    2.669441    2.669441    0.889814
  6    1.779627    1.779627    0.000000
  6    2.669441    0.889814    2.669441

E=  -79.9622 eV
box:   3.559254 0 0 0   3.559254 0 0 0   3.559254
Kpoints: 12 12 12
basis: NGX=  24 NGY=  24 NGZ=  24 NGXF=  48 NGYF=  48 NGZF=  48
```
